# Age-associated insolubility of parkin in human midbrain is linked to redox balance and sequestration of reactive dopamine metabolites

**DOI:** 10.1007/s00401-021-02285-4

**Published:** 2021-03-10

**Authors:** Jacqueline M. Tokarew, Daniel N. El-Kodsi, Nathalie A. Lengacher, Travis K. Fehr, Angela P. Nguyen, Bojan Shutinoski, Brian O’Nuallain, Ming Jin, Jasmine M. Khan, Andy C. H. Ng, Juan Li, Qiubo Jiang, Mei Zhang, Liqun Wang, Rajib Sengupta, Kathryn R. Barber, An Tran, Doo Soon Im, Steve Callaghan, David S. Park, Stephanie Zandee, Xiajun Dong, Clemens R. Scherzer, Alexandre Prat, Eve C. Tsai, Masashi Takanashi, Nobutaka Hattori, Jennifer A. Chan, Luigi Zecca, Andrew B. West, Arne Holmgren, Lawrence Puente, Gary S. Shaw, Gergely Toth, John M. Woulfe, Peggy Taylor, Julianna J. Tomlinson, Michael G. Schlossmacher

**Affiliations:** 1 Program in Neuroscience, Ottawa Hospital Research Institute, Ottawa, ON Canada; 2 Graduate Program in Cellular and Molecular Medicine (Neuroscience), Faculty of Medicine, University of Ottawa, Ottawa, ON Canada; 3 BioLegend Inc., Dedham, MA USA; 4 Department of Pathology and Laboratory Medicine, The Ottawa Hospital, Ottawa, ON Canada; 5 Department of Biochemistry, Karolinska Institute, Stockholm, Sweden; 6 Present Address: Amity Institute of Biotechnology, Amity University, Kolkata, West Bengal 700135 India; 7 Department of Biochemistry, University of Western Ontario, London, ON Canada; 8 Department of Neuroscience, Faculty of Medicine, University of Montreal, Montreal, QC Canada; 9 Ann Romney Center for Neurologic Diseases, Brigham and Women’s Hospital, Boston, MA USA; 10 Division of Neurosurgery, Department of Surgery, The Ottawa Hospital, Ottawa, ON Canada; 11 Department of Neurology, Juntendo University School of Medicine, Tokyo, Japan; 12 Department of Pathology and Laboratory Medicine, University of Calgary, Calgary, AB Canada; 13 Institute of Biomedical Technologies, Italian National Research Council, Segrate, Milan, Italy; 14 Department of Neurobiology, Duke University, Durham, NC USA; 15 Department of Pharmacology and Cancer Biology, Duke University, Durham, NC USA; 16 Proteomics Core Facility, Ottawa Hospital Research Institute, Ottawa, ON Canada; 17 Institute of Organic Chemistry, Research Center for Natural Sciences, Budapest, Hungary; 18 University of Ottawa Brain and Mind Research Institute, Ottawa, ON Canada; 19 Division of Neurology, Department of Medicine, The Ottawa Hospital, Ottawa, ON Canada; 20 Department of Cellular and Molecular Medicine, University of Ottawa, Ottawa, ON Canada; 21 Present Address: Hotchkiss Brain Institute, University of Calgary, Calgary, AB Canada

**Keywords:** Young-onset Parkinson disease, Parkinsonism, Parkin, *PRKN/PARK2* gene, Redox chemistry, Dopamine metabolism, Neuromelanin, Anti-oxidant

## Abstract

The mechanisms by which parkin protects the adult human brain from Parkinson disease remain incompletely understood. We hypothesized that parkin cysteines participate in redox reactions and that these are reflected in its posttranslational modifications. We found that in *post mortem *human brain, including in the *Substantia nigra*, parkin is largely insoluble after age 40 years; this transition is linked to its oxidation, such as at residues Cys95 and Cys253. In mice, oxidative stress induces posttranslational modifications of parkin cysteines that lower its solubility in vivo. Similarly, oxidation of recombinant parkin by hydrogen peroxide (H_2_O_2_) promotes its insolubility and aggregate formation, and in exchange leads to the reduction of H_2_O_2_. This thiol-based redox activity is diminished by parkin point mutants, e.g., p.C431F and p.G328E. In *prkn-*null mice, H_2_O_2_ levels are increased under oxidative stress conditions, such as acutely by 1-methyl-4-phenyl-1,2,3,6-tetrahydropyridine toxin exposure or chronically due to a second, genetic hit; H_2_O_2_ levels are also significantly increased in parkin-deficient human brain. In dopamine toxicity studies, wild-type parkin, but not disease-linked mutants, protects human dopaminergic cells, in part through lowering H_2_O_2_. Parkin also neutralizes reactive, electrophilic dopamine metabolites via adduct formation, which occurs foremost at the primate-specific residue Cys95. Further, wild-type but not p.C95A-mutant parkin augments melanin formation in vitro. By probing sections of adult, human midbrain from control individuals with epitope-mapped, monoclonal antibodies, we found specific and robust parkin reactivity that co-localizes with neuromelanin pigment, frequently within LAMP-3/CD63^+^ lysosomes. We conclude that oxidative modifications of parkin cysteines are associated with protective outcomes, which include the reduction of H_2_O_2_, conjugation of reactive dopamine metabolites, sequestration of radicals within insoluble aggregates, and increased melanin formation. The loss of these complementary redox effects may augment oxidative stress during ageing in dopamine-producing cells of mutant *PRKN* allele carriers, thereby enhancing the risk of Parkinson’s-linked neurodegeneration.

**Supplementary Information:**

The online version contains supplementary material available at 10.1007/s00401-021-02285-4.

## Introduction

Bi-allelic mutations in *PRKN*, which encodes parkin, lead to a young-onset, recessive form of Parkinson disease (PD) [39, 42]. Pathology studies of parkin-deficient brains have demonstrated that neuronal loss is largely restricted to the *S. nigra* and *L. coeruleus*, two brainstem nuclei that synthesize dopamine (reviewed in Doherty et al*.* [15]).

Parkin is a principally cytosolic protein. It has been associated with diverse cellular functions, foremost related to its ubiquitin ligase (E3) activity, the control of inflammation signalling, and maintenance of mitochondrial integrity, as mediated through participation in mitophagy and mitochondrial antigen presentation (MITAP) [5, 57–59, 63, 65, 67, 88] (reviewed in Barodia et al*.* [4]). Although mitophagy has recently been shown to be co-regulated by parkin in the developing heart of mice [26], the diverse roles ascribed to parkin function have not yet explained its selective neuroprotection. For example, vertebrate models of genomic *prkn* deletion do not reproduce dopamine cell loss; one exception is the parkin-deficient *Polg* mouse, where mitochondrial DNA mutagenic stress had been added as a second, genetic hit [75]. The general lack of dopamine cell loss in genomic parkin deficiency-based models of vertebrates could be due to compensatory mechanisms [86], a shorter life span of non-human mammals, and possibly, unique aspects of dopamine metabolism in humans. The latter is exemplified by the generation of cytoplasmic neuromelanin in dopamine synthesizing neurons beginning after childhood [110]. Nevertheless, genomic *prkn*-null models have revealed biochemical and structural changes in high energy-producing cells of flies and murine tissues [4, 21, 102], which suggested the presence of elevated oxidative stress [34, 70, 74]. These observations pointed at a contribution of parkin to redox homeostasis in vivo*.*

Redox equilibrium invariably involves cysteine-based chemistry. There, thiols are subjected to oxidative modifications by reactive oxygen-, reactive nitrogen- and reactive electrophilic species (ROS, RNS, RES) [2, 52], some of which are reversible. Proteins irreversibly conjugated by RES, including by electrophilic dopamine radicals, are either degraded or sequestered within inclusions. It is thought that the latter process occurs via lysosomal functions and underlies neuromelanin formation throughout adulthood [83].

Human parkin contains 35 cysteines [42], its murine homologue 34. Of these, 28 cysteines are involved in the chelation of eight zinc ions within four RING domains [31]. Although Cys431 has been identified as critical in catalyzing human parkin’s E3 ligase activity, 6 other cysteines are structurally unaccounted for, including Cys95 located within parkin’s ‘linker’ domain. Several reports have demonstrated the unique sensitivity of parkin to ROS and RES in cells [50, 60, 103]. Further, RNS and sulfhydration can also modify its cysteines, and NO-/NO_2_-modified parkin variants have been described in cells and brain tissue [6–8, 97, 107]. Oxidation of parkin has been linked to both activating (‘gain-of-function’) and detrimental (‘loss-of-function’) outcomes when tested in the context of parkin’s E3 ligase activity in vitro [8, 51, 60, 107].

We found that wild-type parkin is highly oxidized and insoluble in adult human midbrain, leading us to explore non-E3 ligase-mediated, protective functions. Owing to its large number of cysteine-based thiols, we hypothesized: one, that parkin confers neuroprotection by acting as an anti-oxidant molecule in vivo and thereby contributes to redox balance; two, specifically, that parkin directly lowers ROS- as well as RNS-linked stressors and promotes the conjugation of dopamine radicals (RES); and three, we posited that selective neurodegeneration in *PRKN*-linked, autosomal-recessive PD (ARPD) could be partially explained by the absence of parkin-mediated sequestration of toxic metabolites during decades of human ageing.

## Materials and methods

### Tissue collection

All tissues were collected in accordance with Institutional Review Board-approved guidelines. Fresh frozen samples of the cortical human brain from subjects under 50 years of age were acquired through the University of Alabama and the Autism Tissue Program. *Post mortem*, frozen brain samples from frontal cortices were also obtained from the NICHD Brain and Tissue Bank at the University of Maryland. Brain tissues, including midbrain specimens, with short *post mortem* interval (PMI) were obtained from patients diagnosed clinically and neuropathologically with multiple sclerosis (MS) according to the revised 2010 McDonald’s criteria [76]. There, tissue samples were collected from MS patients, as approved by the Montreal-based CRCHUM research ethics committee. Autopsy samples were preserved and lesions classified using Luxol Fast Blue/Haematoxylin and Eosin staining and Oil Red-O staining, as previously published [14, 48]. No inflamed tissue areas were used in the current study. Additional, fresh-frozen and paraffin-embedded human samples were obtained from the Neuropathology Service at Brigham and Women’s Hospital in Boston, MA and from archived autopsy specimens in the Department of Pathology and Laboratory Medicine of The Ottawa Hospital, Ottawa, ON. Human spinal cord and muscle tissues were collected *post mortem* from organ donors at The Ottawa Hospital with approval from the Ottawa Health Science Network Research Ethics Board.

### Animal tissues

All animal protocols were approved by the review board of the Animal Care and Veterinary Services at the University of Ottawa. Brains were collected from wild-type C57Bl/6J mice from Jackson laboratories (Bar Harbor, ME); *prkn*-null mice were from Dr. Brice’s laboratory [34] and back-crossed onto a pure C57Bl/6J background; *Sod2* ± mice were from Jackson laboratories (pure C57Bl/6J background), and the bi-genic mouse (*prkn*^−/−^//*Sod2*^±^) was created by crossing *prkn*-null mice with *Sod2-*haploinsufficient mice, and the interbreeding of heterozygous offspring. These bi-genic mice have been characterized elsewhere (El-Kodsi et al. in preparation [17]). Following euthanasia by Euthanyl (65 mg/mL) intraperitoneal injection, mouse brains were collected and processed on ice in a Dounce glass homogenizer by 20 passes in Tris salt buffer with vs*.* without the addition of 1% hydrogen peroxide (H_2_O_2_—Sigma), or 0.1–1 M dithiothreitol (DTT—Sigma), transferred to ultracentrifuge tubes and spun during 30 min at 163,202.1 × *g* and 4 °C to extract the soluble fraction. The resulting pellets were further homogenized in the tris-salt buffer with the addition of 2–10% SDS, transferred to ultracentrifuge tubes and spun at 163,202.1 × *g* and 10 °C for 30 min to extract the insoluble fraction. Wild-type mice (of C57Bl/6J or mixed background, as indicated) were used for the analysis of the effects of PMI on murine parkin distribution in the brain. Mice ranging from 4 to 22 months in age were perfused with PBS, their brains collected and processed, as above. Wild-type SAS Sprague Dawley rats were obtained from Charles River Laboratories; frozen frontal lobe specimens of a cynomolgus monkey were provided by the New England Primate Research Center.

### Sequential extraction of parkin from neural tissue

Approximately 1 cm^3^ of the human frontal cortex and midbrain specimens (age range, 5–85 years) were weighed and placed in 3 × volume/weight of Tris-salt buffer (TS; 5 mM Tris, 140 mM NaCl, pH 7.5) containing complete EDTA-free protease inhibitor cocktail, and 10 mM iodoacetamide (IAA, Bio-Rad). The samples were homogenized on ice in a Dounce glass homogenizer by 50 passes, transferred to ultracentrifuge tubes and spun at 163,202.1 × *g* and 4 °C for 30 min. The TS supernatant was transferred to a fresh tube and the pellet was extracted further with the addition of 3 × volume/weight of Triton X-100 buffer (TX, TS + 2% Triton X-100). The samples were mixed by vortexing, incubated on ice for 10 min and centrifuged again using the same setting. The TX supernatant was transferred to a fresh tube and the pellet was extracted further with the addition of 3 × volume/weight of SDS buffer (SDS, TS + 2% SDS). The samples were mixed by vortexing, incubated at room temperature for 10 min and centrifuged again at 163,202.1 × *g* and 12 °C for 30 min. The SDS supernatant was transferred to a fresh tube and the pellet was either stored at − 80 °C or extracted further with the addition of 3 × volume/weight of 6 × non-reducing Laemmli buffer (LB, 30% SDS, 60% glycerol, 375 mM Tris; pH 6.8;), mixed by vortex and incubated at room temperature for 10 min. Samples were centrifuged again at 163,202.1 × *g* and 12 °C for 30 min and the LB supernatant was transferred to a fresh tube. Extracted proteins from TS, TXS and SDS buffers including pellet (20–30 μg) and 10–20 μL of LB extracts were run on SDS-PAGE using reducing (100 mM DTT) and/or non-reducing (0 mM DTT) LB. Following transfer to membranes, Ponceaus S staining (Sigma) was used to probe for equal loading; following washing, membranes were immunoblotted for the detection of parkin (Biolegend 808503, 1: 5000), DJ-1 (Ab18257, 1: 2000), α-synuclein (syn-1, 1:1000 or MJFR-1, 1:2000), LC3B (3868, 1:2000), VDAC (MSA03, 1:5000), MnSOD and GLO1 (each at 1:1000), calnexin (MAB3126, 1:1000), cathepsin D (sc-6486, 1:1000), GRP75 (sc-1058, 1:1000). ImageJ software (version 1.52 k; National Institutes of Health, USA) was used for signal quantification purposes.

### mRNA analyses

*PRKN* mRNA isolated from individual *S. nigra* dopamine neurons, cortical pyramidal neurons and non-neuronal, mononuclear cells from venous blood were processed, as described [16] and annotated in the Human BRAINcode database (www.humanbraincode.org).

### 1-methyl-4-phenyl-1,2,3,6-tetrahydropyridine (MPTP) treatment

Eight to 12 months-old wild-type and *prkn*-null mice were injected intraperitoneally with 40 mg/kg of saline or MPTP and sacrificed an hour later [3]. Brains were harvested for ROS measurement, protein analysis by Western blotting and immunoprecipitation of parkin followed by MS analysis. For LC–MS/MS, murine brains were first incubated in IAA prior to homogenization and fractionation, as described above. Brain homogenates were then incubated with anti-parkin conjugated to magnetic beads (Dynabeads Coupling Kit; Invitrogen), as below. A magnet was used to enrich mouse parkin bound to Prk8 conjugated to beads, and several washes were used to remove non-specific proteins. Eluted fractions (IP elute) along with controls (input, unbound, wash and recombinant parkin protein standards) were run on SDS/PAGE under reducing conditions and blotted with anti-parkin. A sister gel was stained with Coomassie, as described above, and gel slices corresponding to band sizes at 50–75 kDa were excised and analyzed by LC–MS/MS, as described below.

### Recombinant protein expression using a pET-SUMO vector

Plasmid cDNA encoding for wild-type and truncated (amino acid 321-465) human parkin proteins were expressed as 6His-Smt3 fusion proteins in *Escherichia coli* BL21 (DE3) Codon-Plus RIL-competent cells (C2527, New England Biolabs), as previously described [1, 49, 91]. Plasmids encoding for human parkin with p. C95A, p.G328E and p.C431F substitutions were generated with the use of a restriction-free cloning strategy [96] using the following primers: for p.C95A *PRKN* forward: CAGAAACGCGGCGGGAGGCgcTGAGCGGGAGCCCCAGAGCT and *PRKN* reverse: CATCCCAGCAAGATGGACCC; for p.G328E *PRKN* forward: TCCAAACCGGATGAGTGGTG and *PRKN* reverse: CGGGGGCATAACACGCCCcCCATCTGCAGGACACACTC; for p.C431F *PRKN* forward: CTACTCCCTGCCTTGTGTGG and *PRKN* reverse: GCGGACACTTCATGTGCATaaaGCCTCCATTTTTTTCCACTGG.

*DJ-1* and *SNCA* coding regions were cloned from pcDNA3.1 into the pET-SUMO vector using PCR and restriction enzymes. A new restriction site for NotI was inserted between SUMO cleavage site and protein start codon in pET-SUMO using the following primers: *pET-SUMO* forward: GTGATGCCGGCCACGATGCGTCCGGC and *pET-SUMO* reverse: TTTTAAGCTTCCgcggccgcCACCACCAATCTGTTC. The inserts containing wild-type *DJ-1* and *SNCA* sequences with 5′ NotI and 3′ HindIII restriction sites were generated using the following primers and inserted into pET-SUMO using standard conditions: for *DJ-1* forward: agggcggccgcATGGCTTCCAAA and *DJ-1* reverse: cctaagcttCTAGTCTTTAAGAACAAGTGGAGCCTTC; for *SNCA* forward: agggcggccgcATGGATGTATTCATGAAAGG and *SNCA* reverse: ctTTAAGCTTCAGGTTCGTAGTCTTGATACCCTTCAGA.

Quality control steps were performed at the Sequencing Core Facility of the Ottawa Hospital Research Institute (OHRI) to confirm the correct sequences.

Transformed bacteria were grown at 37 °C in 2% Luria Broth containing 30 mg/L kanamycin. All parkin protein-expressing cultures were supplemented with 0.5 mM ZnCl_2_. Protein expression was induced at 16 °C with isopropyl β-d-1-thiogalactopyranoside (Sigma) using 25 μM for wild-type parkin, and 0.75 mM for truncated parkin, DJ-1, α-synuclein and ulp1 protease. Bacteria were harvested after 16–20 h by centrifugation and resuspended in isolation buffer, T500i (50 mM Tris, 500 mM NaCl, 250 μM TCEP, 25 mM imidazole, pH 7.5). Lysozyme (0.1 mg/mL, except for ulp1 protease) treatment and sonication steps (Sonics Vibra Cell) were used to lyse cells. Proteins were collected after 1 h incubation at 4 °C with Ni–NTA agarose and washed several times with buffers T500i and T200i (50 mM Tris, 200 mM NaCl, 250 μM TCEP, 25 mM imidazole, pH 7.5). Fractions of elution buffer T200e (50 mM Tris, 200 mM NaCl, 250 μM TCEP, 250 mM imidazole, pH 7.5) were combined with 2–2.5 mg of 6xHis-tagged ulp1 protease and subsequently dialyzed (6–8 kDa cut-off,) against T200 (50 mM Tris, 200 mM NaCl, 250 μM TCEP, pH 7.5) for 24 h at 4 °C. Remaining proteins were incubated with Ni–NTA agarose for 1 h at 4 °C. Fractions were collected until no protein was detectable, pooled and concentrated to 1 mg/mL using 10 kDa cut-off centrifugation filters (Millipore). The purity and correct masses of isolated proteins were assessed using electron spray ionization mass spectrometry (Agilent 6538 Q-TOF).

### Protein staining methods

All proteins were separated on pre-cast 4–12% Bis–Tris SDS-PAGE gels (NPO321BOX, NPO322BOX, NPO336BOX) from Invitrogen using MES running buffer (50 mM MES, 50 mM Tris, 1 mM EDTA and 0.1% SDS, pH 7.3) and Laemmli loading buffer (10% SDS, 20% glycerol, 0.1% bromophenol blue, 0.125 M Tris HCl, 200 mM DTT or β-mercaptoethanol). Proteins were stained in gel using SilverQuest™ Silver Staining Kit (LC6070) from Invitrogen or Coomassie brilliant blue R-250 dye (20,278) from Thermo Scientific using the following protocol: The gel was transferred to a plastic container and rocked for 30 min in Fix Solution (10% acetic acid, 50% methanol), followed by staining for 2–24 h (0.25% Coomassie R250) until the gel turned a uniform blue. The stain was replaced with Destain Solution (7.5% acetic acid and 5% methanol) and the gel was rocked until crisp blue bands appeared. Following a wash with water, the gel was stored in 7% acetic acid. Proteins transferred to PVDF (Bio-Rad) membranes were stained with Ponceau S solution (Sigma) for 20 min, washed three times with water, imaged and then destained with 0.1 M NaOH prior to Western blotting.

### Dynamic light scattering assay

For each recombinant protein preparation tested, the buffer (50 mM Tris, 200 mM NaCl and 250 μM TCEP, pH 7.5) was exchanged for a 20 mM phosphate buffer with 10 mM NaCl (pH 7.4). 20 μM full-length wild-type recombinant parkin was centrifuged at 21,000 × *g* for 60 min at 4 °C and light scattering intensity of the supernatant was collected 30 times at an angle of 90° using a 10 s acquisition time. Measurements were taken at 37 °C using a Malvern Zetasizer Nano ZS instrument equipped with a thermostat cell. The correlation data were exported and analyzed using the nanoDTS software (Malvern Instruments). The samples were measured at 0, 1, 3 and 5 h. Following 24 h incubation, 2 mM DTT was added to the sample and the light scattering intensity of the supernatant was measured again.

### Far UV circular dichroism spectroscopy

Fifteen μM of reduced and partially oxidized full-length wild-type, recombinant (r-) parkin was measured at *t* = 0 and *t* = 5 days of incubation under native conditions in 20 mM phosphate, 10 mM NaCl buffer. The aggregate-rich phase and the monomer-rich phase in the samples were separated with ultracentrifugation (100,000 × *g* for 2 h). Far UV circular dichroism (CD) spectra were recorded for the monomer- and aggregate-rich phase of protein samples using a JASCO J-720 spectrometer. The final spectrum was taken as a background-corrected average of 5 scans carried out under the following conditions: wavelength range 250–190 nm at 25 °C; bandwidth was 1 nm; acquisition time was 1 s and intervals was 0.2 nm. Measurements were performed in a 0.01 cm cell. CD spectra were plotted in mean residue molar ellipticity units (deg cm^2^ dmol^−1^) calculated by the following equation: [Θ] = Θ_obs_/(10*ncl)*, where [Θ] is the mean residue molar ellipticity as a function of wavelength, Θ_obs_ is the measured ellipticity as a function of wavelength (nm), *n* is the number of residues in the protein, *c* is the concentration of the protein (M), and *l* is the optical path length (cm). Secondary structure analysis of proteins using CD spectroscopic data was carried out using the BeStSel (Beta Structure Selection) software [41, 61, 62, 90].

### Cysteine labeling for mass spectrometry

Recombinant protein samples were first prepared by exchanging the T200 buffer for PBS. The protein concentrations were measured and adjusted to 10 μM using PBS. Stock solutions of 500 mM DTT, 100 mM IAA, 100 mM H_2_O_2_ and 250 mM EDTA were prepared in PBS. A stock of 500 mM NEM was prepared in ethanol immediately before use. The stepwise Cys labeling procedure was as follows: A 10 μL aliquot of protein (at 10 μM) was reacted with hydrogen peroxide at various concentrations, as indicated (Supplementary Table 2, online resource) for 30 min at 37 °C as indicated. Any unreacted cysteines were alkylated with incubation with 5 mM IAA (either with or, in some runs, without 10 mM EDTA) for 2 h at 37 °C. Previously oxidized cysteines were then reduced by treatment with 40 mM DTT for 30 min at 37 °C. Newly reduced cysteines were alkylated by incubation with 85 mM *N*-ethyl maleimide (NEM) for 2 h at 37 °C. The samples were separated on SDS-PAGE using Laemmli buffer containing 100 mM DTT and proteins visualized using Coomassie staining. Appropriate bands were excised and analyzed by liquid chromatography mass spectrometry (LC–MS/MS).

### Protein identification by LC–MS/MS

Proteomic analyses were performed at the OHRI Proteomics Core Facility (Ottawa, Canada). Proteins were digested in-gel using trypsin (Promega) according to the method of Shevchenko [84]. Peptide extracts were concentrated by Vacufuge (Eppendorf). LC–MS/MS was performed using a Dionex Ultimate 3000 RLSC nano HPLC (Thermo Scientific) and Orbitrap Fusion Lumos mass spectrometer (Thermo Scientific). MASCOT software version 2.6.2 (Matrix Science, UK) was used to infer peptide and protein identities from the mass spectra. For detection of dopamine metabolites on parkin, the following variable modifications were included: 5,6-indolequinone (+ C_8_O_2_NH_3_, m/z shift + 145), aminochrome (+ C_8_O_2_NH_5_, + 147), aminochrome + 2H (+ C_8_O_2_NH_7_, + 149), and dopamine quinone (+ C_8_O_2_NH_9_, + 151). These samples were prepared for analysis without any use of DTT or IAA. The observed spectra were matched against human sequences from SwissProt (version 2018-05) and also against an in-house database of common contaminants. The results were exported to Scaffold (Proteome Software, USA) for further validation and viewing.

Analysis of the r-parkin holoprotein and of three runs of H_2_O_2_-exposed r-parkin (Supplementary Table 2, online resource) was also performed at the University of Western Ontario. There, samples were run on a QToF Ultima mass spectrometer (Waters) equipped with a Z-spray source and run in positive ion mode with an Agilent 1100 HPLC used for LC gradient delivery (University of Western Proteomics Facility).

### MaxQuant analysis of mass spectrometry data

For select experiments, the raw MS data files were further processed with MaxQuant software version 1.6.5 and searched with the Andromeda search engine [10]. The reference fastas were set to uniprot-human (version 2019-02-12) and uniprot-ecoli. The *E. coli* proteome was included to account for bacterial proteins present in the recombinant protein samples. The ‘second peptides’ and ‘match between runs’ settings were enabled. All other settings were left as default. Selected variable modifications included oxidation (Met), acetylation (protein N-terminus), and carbamidomethyl (Cys), as well as custom modifications for pyro-carbamidomethyl (N-terminal Cys), N-ethylmaleimide (Cys), and NEM + water (Cys). For data analyses, site-level intensity values were obtained from the MaxQuant-generated “CarbamidomethylSites” table which combines the intensity of MS1 signals from all peptides covering a particular cysteine residue.

### Immunoprecipitation (IP) of brain parkin

Conjugation of anti-parkin antibody (Prk8, 808503, lot B209868) and clone A15165-B (this report: Suppl. Figure 8c) to magnetic beads at a final concentration of 10 mg of antibody/mL of beads was carried out following the Magnetic Dynabeads Antibody Coupling Kit from Invitrogen (14311D). Human tissue lysates were also prepared using the sequential extraction of proteins from neural tissue protocol, as described above, with the addition of 10 mM IAA prior to homogenization. Equal amounts of protein from TS tissue extracts (*n* = 4) and SDS tissue extracts (*n* = 8) were diluted in TS buffer, resulting in final SDS concentrations of 0.0175–0.05% in the SDS extracts. For the IP, anti-parkin primary antibody-conjugated agarose beads were first prepared by multiple washes with 1 mL of TS buffer using centrifugation (1000 × *g* at 4 °C for 3 min) and adhesion to a strong magnet. The amount of Prk8 conjugated agarose beads used for each experiment were approximated based on the amount of parkin (μg)/sample calculated by densitometry when the sample was compared to recombinant parkin protein standards using Western blotting with Prk8 as a primary antibody. The mixture was incubated for 16 h at 4 °C with slow rotation. Unbound proteins, were separated from the beads by centrifugation (1000 × *g* at 4 °C for 3 min) followed by adhesion to a strong magnet and saved as the IP “unbound” fraction.

Beads were washed three times with 1 mL of ice-cold RIPA buffer (1% nonionic polyoxyethylene-40, 0.1% SDS, 50 mM Tris, 150 mM NaCl, 0.5% sodium deoxycholate, 1 mM EDTA) using centrifugation (1000 × *g* at 4 °C for 3 min) and adhesion to a strong magnet. Approximately 5–10 µL of each wash was combined and saved as the IP “wash” fraction. To elute antibody-bound proteins, 35 µL of 6X reducing Laemmli buffer (30% SDS, 60% glycerol, 0.3% bromophenol blue, 0.375 M Tris, 100 mM DTT, pH 6.8) was added to the beads and the samples were boiled for 5 min. Following centrifugation (1000 × *g* at 4 °C for 3 min), the supernatant was transferred to a fresh tube labeled “IP elute” and the beads were discarded. To assess IP efficiency, eluted fractions (IP elute), along with controls (input, unbound, wash and recombinant parkin protein standards) were run on SDS/PAGE and blotted with anti-parkin antibody (Prk8, MAB5512 Millipore or 2132S Cell Signalling). Human IP elutes used for mass spectrometry (MS) analysis were incubated with 500 mM NEM (as indicated for select runs) for 16 h at 4 °C prior to SDS-PAGE and further processed for MS, as described above. Gel slices corresponding to band sizes at 50–53 kDa were excised and analyzed by LC–MS/MS.

### Reactive oxygen species (H_2_O_2_) measurements in recombinant protein preparations, cell lysates and tissue homogenates

An Amplex^®^ Red hydrogen peroxide/peroxidase assay kit (Invitrogen A22188) was used to monitor endogenous levels of H_2_O_2_ in aliquots of tissues and cells, and in test tubes following either exposure to increasing concentrations of H_2_O_2_, *n*-ethylmaleidmide (NEM), and ethylenediaminetetraacetic acid (EDTA), or after incubation with either select, recombinant parkin proteins, or DJ-1, α-synuclein, bovine serum albumin (Thermo Scientific), ring finger protein 43 (RNF43—BioLegend, MA.), HOIL-1-interacting protein (HOIP—Boston Biochem, MA.), glutathione (Sigma), or catalase (Sigma) for 30 min at room temperature. Pre-weighed cortex pieces from the human brain and pelleted cells were homogenized on ice in the 1 × reaction buffer provided (Invitrogen A22188) using a Dounce homogenizer (3 × volume to weight ratio). Homogenates were diluted in the same 1 × reaction buffer (fivefold to tenfold). A serial dilution of the H_2_O_2_ standard provided was prepared (20, 10, 2 and 0 μM). Fifty μL of standards and samples were plated in a 96-well black plate with a clear flat bottom (Thermo Fisher Scientific). The reaction was started by the addition of 50 μL reaction buffer, Amplex^®^ Red and horseradish peroxidase (HRP) (10 mM Amplex^®^ Red and 10 U/mL HRP). Plates were incubated at room temperature for 30 min protected from light. A microplate reader was used to measure either fluorescence with excitation at 560 nm and emission at 590 nm, or absorbance at 560 nm. The obtained H_2_O_2_ levels (μM) were normalized to the tissue weight (g) or protein concentration (μg/μL). The same assay was also used to measure parkin and glutathione’s peroxidase activity compared to horseradish peroxidase.

### Chemiluminescence-based, direct reactive oxygen species assay

The assay was modified from Muller et al. [64] to measure the ROS-quenching ability of parkin proteins, DJ-1, GSH, and catalase. Protein concentrations were quantified using Bradford assay and adjusted to 5, 10, 15 and 30 μM in buffer not containing TCEP. Bovine serum albumin (BSA; 10 and 20 μM; Thermo Scientific), glutathione (15, 20, 200, 400, 800 and 2000 μM; Sigma), and catalase (15 μM, Sigma) were prepared. Stock solutions of H_2_O_2_ for standard curve were prepared at 5, 10, 20, 40 and 50 mM in 0.1 M Tris HCl pH 8.0 using 30% H_2_O_2_. Stock solutions of 300 mM luminol and 40 mM 4-iodophenol were prepared in DMSO and protected from light. Signal reagent, containing 1.94 mM luminol (Sigma) and 0.026 mM 4-iodophenol (Sigma), was prepared in 0.1 M Tris HCl pH 8.0 and protected from light. A 0.4% horseradish peroxidase solution was prepared using HRP-linked anti-rabbit secondary antibody diluted in Stabilizyme solution (SurModics SZ02). Each read was set up in triplicate on a white polystyrene 96-well plate (Thermo Scientific 236,105) and to each well was added 80 μL Stabilizyme, 15 μL of 0.4% horseradish peroxidase (HRP) and 25 μL of sample or controls. One of the injectors in a Synergy H1Multi-Mode Plate Reader (Bio Tek) was primed and set to inject 15 μL of signal reagent and 15 μL of each H_2_O_2_ stock solution was manually added to corresponding controls and samples just prior to reading. Final concentrations of reagents were 0.04% HRP, 500, 1000, 2000, 4000 and 5000 μM H_2_O_2_, 194 μM luminol, 2.6 μM 4-iodophenol and 0.8, 1.7, 2.5 or 5 μM of protein. The plate reader was set to measure luminescence every 1 min for a total of 10 min.

The resulting kinetic data were converted to the area under the curve (AUC) using Prism software version 6. For samples pre-incubated with 20 mM iodoacetamide, a stock solution of 1 M iodoacetamide was prepared. To each well containing 25 μL of the sample, 0.52 μL of 1 M iodoacetamide and 0.48 μL of buffer not containing TCEP was added and the samples were incubated for 2 h at 37 °C. Following incubation, the reagents for chemiluminescence were added as above except 79 μL of Stabilizyme was used instead of 80 μL and the samples were analyzed as above.

### Thiol quantification in recombinant proteins

Recombinant protein samples were first prepared by exchanging the T200e protein buffer (50 mM Tris, 200 mM NaCl and 250 μM TCEP, pH 7.5) for T200 using repeat centrifugations (8 times 4000 × *g* at 4 °C for 10 min) in Amicon Ultra 10 kDa MWCO filters (Millipore). The protein concentrations were measured and recorded. A glutathione stock solution of 32,539 μM was prepared by dissolving 1 mg glutathione (GSH) in 1 mL of T200 and the standards 0, 50, 101, 203, 406, 813 and 1000 μM were prepared by serial dilution in T200. The reaction buffer (0.1 M sodium phosphate, pH 8.0) was prepared by adding 93.2 mL 1 M Na_2_HPO_4_ and 6.8 mL of NaH_2_PO_4_ in 1 L of water. Thiol detecting reagent (Ellman’s reagent) was prepared by dissolving 2 mg of 5,5′-dithio-bis-[2-nitrobenzoic acid] (DNTB; Sigma) in 1 mL of reaction buffer. The assay was performed in 96-well clear round-bottom plates (Corning) by adding 50 μL of thiol detecting reagent to 50 μL of sample or standard and incubating for 15 min at room temperature.

The resulting 5-thio-2-nitrobenzoic-acid (TNB) produced was measured by absorbance at 412 nm using a Synergy H1Multi-Mode Plate Reader (Bio Tek). The amount of free thiols detected in each sample was calculated using the regression curve obtained from the glutathione standards and dividing by the concentration of the sample.

### Zinc ion release assay

A zinc quantification kit (Abcam—ab102507) was used to assay zinc ion (Zn^2+^) release from proteins. Recombinant human proteins (wild-type r-parkin and r-DJ-1) were spun in 10 or 30 kDa cut-off centrifugation filters (Millipore) to remove residual TCEP. Increasing concentrations of protein (0 to 2.5 μM) were incubated under basal conditions or with the addition of H_2_O_2_ (2 mM) or DTT (100 mM) for 30 min at 37 °C. A standard curve was prepared using a zinc standard (stock, 50 mM) provided by the manufacturer. Two hundred μL of the reaction mixture was added to 50 μL standards and samples on 96 well plates (Thermo Fisher Scientific) followed by incubation at room temperature for 10 min. A microplate reader was used to measure the absorbance at OD560 nm. The background was corrected by subtracting the value derived from wells of zero zinc standard from all readings.

### Cell cytotoxicity assay

Human neuroblastoma cell lines (M17) without transduction (controls), or transduced by vector-only plasmid (Myc-tag), or those with low levels of stable expression of Myc-parkin cDNA (P5) and high levels of stable expression of Myc-parkin (P17), or sister lines transiently over-expressing flag-parkin (wild-type), flag-vector and flag-parkin carrying one of three-point mutations (p.C431F; p.G3289E; p.C95A) were grown in 6-well culture plates at 0.3 × 10^6^ cell density (80% confluence). There, Opti-MEM media (Gibco 11,052-021) contained heat-inactivated fetal bovine serum (Gibco 10,082–147), Pen/strep/Neo (5 mg/5 mg/10 mg; Gibco 15,640-055), MEM-non-essential amino acids (10 mM; Gibco 11,140-050) and sodium pyruvate (100 mM). For rescue experiments, M17 cells transiently expressing cDNA for flag-vector, flag-parkin wild-type, and variants carrying p.G328E, p.C431F, or p.C95A-encoding parkin protein were used. There, 4 μg of cDNA was transfected using a 1:1 ratio of cDNA to Lipofectamine 2000 (52,887, Invitrogen) in OPTI-MEM transfection medium. Lipofectamine 2000 and cDNA were first incubated for 20 min at room temperature before being applied directly to the cells for 1 h at 37 °C with 5% CO_2_ followed by direct addition of fresh growth medium. The cells were incubated another 20–24 h at 37 °C with 5% CO_2_.

Dopamine hydrochloride (Sigma) 200 mM stock was prepared. Cells were washed with fresh media once and then incubated with media alone or supplemented with dopamine at final concentrations of 20 μM and 200 μM for 18–20 h. Post dopamine exposure, conditioned media were collected for cytotoxicity assays and cells were harvested for lysis in TS buffer, vortexed and centrifuged. Supernatants were collected and saved for Western blot analyses and to be assessed for cytotoxicity. Cell pellets were suspended in 2–10% SDS buffer and centrifuged to collect the ‘insoluble fraction’.

Vybrant ™ cytotoxicity assay kit (Molecular Probes V-23111) was used to monitor cell death through the release of the cytosolic enzyme glucose 6-phosphate dehydrogenase (G6PD) by damaged cells into the surrounding medium. Fifty μl of fresh media (without any exposure to cells) as well as conditioned media from control and stressed cells, in addition to lysates of M17 cells (as a positive control for maximum G6PD), were added to a 96-well microplate. Fifty μl of reaction mixture containing buffer (as per manufacturer) and resazurin, which reacts with G6PD generating fluorescently detectable resorufin, were added to each well, and the mircroplate was incubated at 37 °C for 30 min. A microplate reader was used to measure either fluorescence with excitation at 560 nm and emission at 590 nm, where the rise in fluoresence indicates a rise in G6PD levels as a surrogate marker of cell death.

### Aminochrome synthesis

A solution of 0.1 M sodium phosphate buffer pH 6.0 was prepared from a mixture of 12 mL of 1 M NaH_2_PO_4_ and 88.0 mL of 1 M Na_2_HPO_4_. The reaction buffer (0.067 M sodium phosphate, pH 6.0) was prepared by adding 33 mL of 0.1 M sodium phosphate buffer to 17 mL water. A solution of 10 mM dopamine in reaction buffer was prepared by adding 19 mg of dopamine hydrochloride (Sigma) to 1 mL of reaction buffer. Oxidation was activated by adding 5 μL of tyrosinase (25,000 U/mL; Sigma) and the mixture was incubated at room temperature for 5 min. Tyrosinase was separated from the oxidized dopamine using a 50 kDa cut-off Amicon Ultra centrifugation filter (Millipore) by centrifuging at 21,000 × *g* for 10 min. The absorbance of the filtrate was measured at a wavelength of 475 nm (Ultrospec 2100 pro spectrophotometer, Biochrom) and the concentration of aminochrome was determined using the Beer-Lambert equation and extinction coefficient of 3058 L × mol^−1^ × cm^−1^.

### Redox chemistry reactions including oxidation of cysteine-containing proteins in vitro

Purified, recombinant proteins were prepared by removing excess TCEP, present in the elution buffer, by using repeat centrifugations (8 times 4000 × *g* at 4 °C for 10 min) in Amicon Ultra 10 kDa MWCO filters (Millipore). Protein concentrations were measured and adjusted to 20 μM. Stock solutions of hydrogen peroxide (H_2_O_2_, 9.8 mM) and aminochrome (as described above) and used at concentrations of 0-200 μM, were prepared. An aliquot of 10 μL of each protein sample (at 20 μM) was reacted with oxidants at the following concentrations: 0, 20, 200, 500, 750, 1000, 2000 μM for H_2_O_2_, and 0, 10 µM, 100 µM, 1 mM, 10 mM, 100 mM, 1000 mM for DTT. Samples were treated for 30 min at 37 °C and centrifuged at 21,000 × *g* for 15 min. The supernatant was transferred to a fresh tube and the remaining pellet was extracted with 10 μL of T200 containing either 10% SDS or 100 mM DTT. The pellets were incubated again for 30 min at 37 °C and centrifuged at 21,000 × *g* for 15 min. Laemmli buffer (10 μL, containing 100 mM mercaptoethanol) was added to both the pellet and supernatant fractions and samples were separated by SDS-PAGE and visualized by silver staining. Specific bands of aminochrome-treated wild-type, full-length r-parkin were excised and analyzed by LC–MS/MS, as described above.

### In vitro melanin formation assay

Recombinant protein samples were first prepared by exchanging the T200e protein buffer (50 mM Tris, 200 mM NaCl and 250 μM TCEP, pH 7.5) for T200 (50 mM Tris and 200 mM NaCl, pH 7.5) using repeat centrifugations (8 times 4000 × *g* at 4 °C for 10 min) in Amicon Ultra 10 kDa MWCO filters (Millipore). The protein concentrations were measured and adjusted to 20 μM using T200. A 0.067 M sodium phosphate buffer, pH 6.0, was prepared by adding 33 mL of 0.1 M sodium phosphate buffer to 17 mL water and adjusting the pH using HCl. A stock solution of 100 mM dopamine hydrochloride was prepared in 0.067 M sodium phosphate buffer and stock solutions of 100 mM reduced glutathione and H_2_O_2_ were prepared in T200.

Samples and controls were prepared in 100 μL total volume that contained 10 μL of protein sample or T200, 10 μL of 100 mM dopamine or 0.067 M sodium phosphate buffer, 10 μL of 100 mM glutathione or T200 buffer, and 70 μL T200. Unless otherwise indicated, the final concentration of protein was 2 μM and the final concentration of reagents was 10 mM. The samples and controls were plated in triplicate, and absorbance read at 405 and 475 nm every 90 s for 1 h and up to 4 h (Synergy H1Multi-Mode Plate Reader; Bio Tek).

### Immunohistochemistry

Immunohistochemistry (IHC) was performed on paraffin-embedded sections, as previously described [81, 82, 87]. Briefly, prior to antibody incubation, sections were deparaffinized in xylene and successively rehydrated through a series of decreasing ethanol concentration solutions. Endogenous peroxidase activity was quenched with 3% hydrogen peroxide in methanol, followed by a standard citric acid-based antigen retrieval protocol (CitriSolv, Decon Labs) to unmask epitopes. Sections were blocked in 10–20% goat serum in PBS-Tween (Tween 20 0.075%) to reduce non-specific signal. Sections were incubated overnight at 4 °C in primary antibodies diluted in 1–5% goat serum in PBS-T according to the following concentrations: novel anti-parkin mAbs from Biolegend clones D (BioLegend, A15165-D; 1:250), clone E (BioLegend, A15165E; 1:2,000), and clone G (1:250), Prk8 (BioLegend, MAB5512; 1:500) as well as anti-LAMP-3/CD63 (Santa Cruz, SC5275; 1:100), anti-LC3B (Sigma, L7543-200uL; 1:100), anti-VDAC (MitoScience, MSA03; 1:100). Biotinylated, secondary antibodies (anti-mouse IgG (H + L), Vector Labs, BA-9200, and biotinylated anti-rabbit IgG (H + L), both made in goat; Vector Labs, BA-1,000) were diluted to 1:225, and sections were incubated for 2 h at room temperature. The signal was amplified with VECTASTAIN® Elite® ABC HRP Kit (Vector Labs, PK-6100), and visualized via standard diaminobenzidine solution (DAB, 55 mM), or Vina green (Biocare Medical, BRR807AH), or most frequently by using ‘metal enhanced DAB’ (Sigma, SIGMAFAST™ DAB with Metal Enhancer D0426). Samples were counterstained with Harris Modified Hematoxylin stain and dehydrated through a series of increasing ethanol concentration solutions and xylene. Permount (Fisher Scientific, SP15-100) was used for mounting and slides processed for IHC were visualized and processed using a Quorum Slide Scanner at the OHRI Imaging Core Facility.

### Immunofluorescence and confocal microscopy

Paraffin-embedded human midbrain sections were stained by routine indirect immunofluorescence (IIF) with the following details. Antigen retrieval was performed in Tris–EDTA buffer pH 9.0 for 10 min. Primary antibodies were incubated overnight at 4 °C. Details for primary antibodies anti-parkin clone E (1:500), anti-LAMP-3 (1:250) are described above. Fourty min-long incubations with the following secondary antibodies were performed: goat anti-mouse alexa fluor 488 (1:200), goat anti-rabbit alexa fluor 594 (1:500). Slides were mounted with fluorescence mounting medium using DAPI. Sections stained for IIF were imaged using a Zeiss LSM 880 AxioObserver Z1 with an Airyscan Confocal Microscope and then processed for further analysis using Zeiss Zen and Fiji software.

### Statistical analyses

Statistical analyses were performed using GraphPad Prism version 6 (GraphPad Software, San Diego, CA, USA, www.graphpad.com). Differences between two groups were assessed using an unpaired *t*-test. Differences among 3 or more groups were assessed using a one-way or two-way ANOVA followed by Tukey’s post hoc corrections to identify statistical significance. Select post hoc tests are depicted graphically to visualize significance. For all statistical analyses, a cut-off for significance was set at 0.05. Data are displayed with *p* values represented as *p < 0.05, **p < 0.01, ***p < 0.001, and ****p < 0.0001. Linear regression (for the continuous dependent variable, e.g*.,* % soluble parkin and H_2_O_2_ concentration) was performed using R version 3.6.0. Furthermore, to address the effect of age on parkin solubility (defined as a dichotomous variable using criteria below), logistic regression and receiver operating characteristic (ROC) curves and area under the ROC curve (AUC) values were calculated using R, as reported [87].

## Results

### Parkin solubility declines in the ageing human brain including in the *Substantia nigra*

**Fig. 1:**
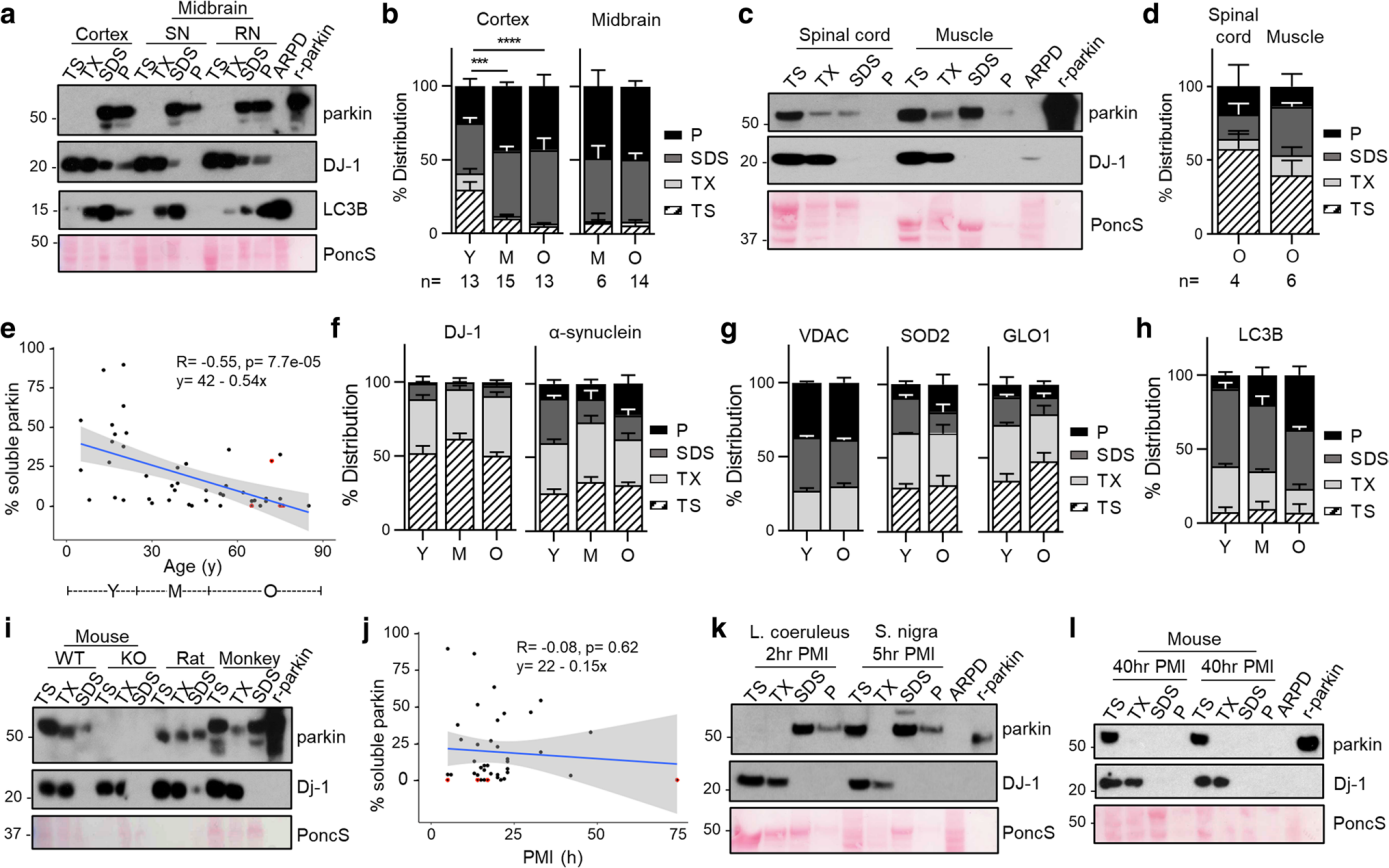
Parkin’s decline in solubility is specific to the adult human brain and correlates with age. **a** Representative Western blots of parkin, DJ-1, and LC3B distribution in human cortex, *S. nigra* (SN) and red nucleus (RN) serially fractionated into Tris-NaCl buffer-soluble (TS), Triton X-100-soluble (TX), 2% SDS-soluble (SDS) extracts and the pellet (P) lysed in 30% SDS-containing buffer. SDS extracts from *PRKN*-linked Parkinson disease (ARPD) brain and recombinant, human parkin (r-parkin) are included. Ponceau S is shown as loading control. **b** Relative distribution of parkin signal within each fraction for cortex and midbrain grouped by age ranges: young (Y ≤ 20 years; *n* = 13); mid (M > 20 years but < 50 years; *n* = 15 for cortex, and *n* = 6 for midbrain); older (O ≥ 50 years; *n* = 13 for cortex and *n* = 14 for midbrain). Data shown as mean ± SEM. The significance in protein distribution between soluble (TS + TX) and insoluble (SDS + pellet) fractions was determined using 2-way ANOVA [*F*(2, 76) = 26.21, *p* < 0.001] with Tukey’s post hoc test (***p < 0.001; ****p < 0.0001). Additional Western blots are shown in Supplementary Fig. 1a–c, online resource. Midbrains include both control and neurological disease cases, as listed in Supplementary Table 1, online resource. **c** Western blots of parkin and DJ-1 as well as Ponceau S staining of serial fractions from the representative human spinal cord and skeletal muscle tissues from individuals ≥ 50 years. **d** Relative distribution of parkin as in (**b**) for human spinal cord (*n* = 4) and skeletal muscle specimens (*n* = 6) from donors aged 50–71 years. **e** Univariate linear regression analysis of parkin solubility in cortices as a function of age (*n* = 46). Each brain is represented by an individual dot; red circles denote three cases of parkinsonism not linked to *PRKN*; the linear regression line (in blue) and 95% confidence intervals (grey) are shown. Age ranges that correspond to Y–O–M in (**b**) are shown under the graph. Age coefficient was − 0.54 (95% CI: − 0.79 to − 0.29, *P* = 7.7e^−05^). **f–h** Relative distribution of **f** DJ-1, α-synuclein and **g** VDAC, MnSOD, glyoxalase (GLO1) and **h** LC3B in human cortices (*n* = 3–5 per age group), as described in (**b**). Representative Western blots are shown in Supplementary Fig. 1b, c, online resource. **i** Western blots of parkin and Dj-1 and Ponceau S staining of serial fractions from whole brains of wild-type (WT; 8 months of age) and *prkn* knock-out (KO) mice, WT rat (WT; 14 months) and from frontal cortex of a cynomolgus monkey (60 months). **j** Univariate linear regression analysis of parkin solubility in human brain as a function of length for *post mortem* interval (PMI; in hours); the linear regression line (in blue) and 95% confidence intervals (grey) are shown (*n* = 41 cortices). **k** Western blots of parkin and DJ-1 distribution in two human brainstem nuclei, *L. coeruleus* and *S. nigra*, which were collected within 2–5 h after death prior to freezing and processed as in (**a**, **c**). **l** Immunoblots for endogenous parkin and Dj-1 as well as Ponceau S staining from serially extracted WT mouse brains (*n* = 3) dissected after a 40 h *post mortem* interval. Note, Western blots shown in this figure followed SDS/PAGE under reducing conditions

Parkin’s biochemistry in the human brainstem *vs.* other regions of the neuroaxis has remained largely unexplored [73]. We serially fractionated 20 midbrain specimens (ages, 26–82 years) and > 40 cortices (ages, 5–85 years) from human subjects, which had been collected *post mortem* (Fig. 1, Supplementary Fig. 1; Supplementary Table 1, online resource). In control brain, we found that before the age of 20 years, nearly 50% of cortical parkin was found in soluble fractions generated by salt [Tris-NaCl; TS]- and non-ionic detergent [Triton X-100; TX]-containing buffers (Fig. 1a, b; Supplementary Fig. 1a, online resource). In contrast, after age 50 years, parkin was predominantly (> 90%) found in the 2% SDS-soluble (SDS) fraction and the 30% SDS extract of the final fractionation pellet (P). The same distribution was seen in adult midbrain (e.g.,* S. nigra*; red nucleus), the pons (*e.g.*, *L. coeruleus*), and the striatum (Fig. 1a, b; Supplementary Fig. 1a–c, online resource).

Intriguingly, in older individuals (ages, ≥ 50 years) approximately half of the detectable parkin remained soluble in the human spinal cord and skeletal muscle specimens, which had also been collected *post mortem* (Fig. 1c, d). We used univariate linear regression analysis to explore a correlation between soluble parkin (of TS- and TX-fractions relative to the total signal for parkin, plotted as %) and age in human control cortices (Fig. 1e). The regression coefficient of age was − 0.54 (at a 95% confidence interval (CI) of − 0.79 to − 0.29, *P* = 7.7e^−05^), where the multiple R-squared value was 0.302. When defining parkin solubility as a binary variable, i.e., the presence or absence of soluble parkin in TS- and TX-fractions (absent defined as less than 2% of total signal), and using logistic regression analysis, we found that the transition to insoluble parkin occurred between the ages of 28 years (with low sensitivity but high specificity values) and 42 years (with high sensitivity and low specificity values) (data not shown).

This age-dependent partitioning of parkin was not seen for any other protein examined, including two other PD-linked proteins, i.e., DJ-1 and α-synuclein (Fig. 1a, f), or for organelle-associated markers, e.g., cytosolic glyoxalase-1, peroxiredoxin-1 and -3; and endoplasmic reticulum-associated calnexin. Notably, mitochondrial markers, e.g., voltage-dependent anion channel (VDAC) and Mn^2+^-superoxide dismutase (MnSOD), also did not partition with parkin (Fig. 1g; Supplementary Fig. 1b, c, online resource; and data not shown). In contrast, parkin did co-distribute with LC3B, a marker of protein aggregation, foremost in brain specimens from older individuals (Fig. 1a, h; Supplementary Fig. 1c, online resource).

The age-associated loss in parkin solubility appeared unique to the human brain in that it remained predominantly soluble in the adult nervous system of other species, *e.g.,* mice and rats as well as cynomolgus monkey, which were processed in the same way (Fig. 1i). Specifically, in brain lysates of two different wild-type strains of mice (C57Bl/6J, and a mixed 129S//FVB/N//C57Bl/6J background) aged to 18 and 22 months respectively, parkin remained present in the soluble fraction throughout their lifespan (Supplementary Fig. 1d, online resource; and data not shown).

In soluble fractions from older humans, we did not detect any truncated species of parkin using several, specific antibodies (data not shown). Despite the loss of parkin solubility with progression in age, *PRKN* mRNA was detectable in individual neurons isolated from the *S. nigra* and cortex throughout all age groups; there, transcript levels in neurons did not correlate with the subject’s age (Supplementary Fig. 1e, f, online resource).

Our analysis comprised samples with *post mortem* intervals (PMI) that spanned from 2 to 74 h (Supplementary Table 1, online resource). Using univariate linear regression analysis, we detected no correlation between parkin solubility in human control cortices (*n* = 41) and PMI length, where the regression coefficient for PMI measured − 0.15 (95% CI: − 0.76 to 0.46, *P* = 0.62), and the multiple R-squared value was 0.0064 (Fig. 1j). As expected, PMI did not correlate with the age of the deceased person (not shown). Likewise, wild-type parkin was found to be largely insoluble in striatal, midbrain and pontine samples isolated from aged subjects with PMIs as short as 2 to 6 h (Fig. 1k; Supplementary Fig. 2a, b, online resource). We further explored a possible contribution of PMI to parkin solubility by mimicking conditions of some of the human autopsy cases, using adult mice. This included a PMI length of up to 40 h, where animals were kept at room temperature for the first 14 h, followed by storage over 26 h at 4 °C before removal of their brain; in these cases, parkin remained in the soluble compartments (Fig. 1l and data not shown). While we cannot exclude that PMI length could affect parkin’s solubility in some cases, the age-dependent loss of parkin solubility observed in human brain samples of our cohort was not due to the PMI.

Further, we determined that the decline in detectable parkin solubility in the aged human brain did not differ based on the sex of the deceased person, such as when examined by univariate linear regression analysis or by multivariate analysis (data not shown); it was also not caused by either tissue freezing prior to protein extraction or the pH value of the buffer (Supplementary Fig. 2c–f, online resource). Moreover, employing the commonly used ‘RIPA buffer’ instead of our serial extraction buffers resulted in the release of parkin into the supernatant with some reactivity left in the pellet, as expected (Supplementary Fig. 2g, online resource).

### Decline in parkin solubility correlates with rising hydrogen peroxide levels in the mammalian brain

**Fig. 2:**
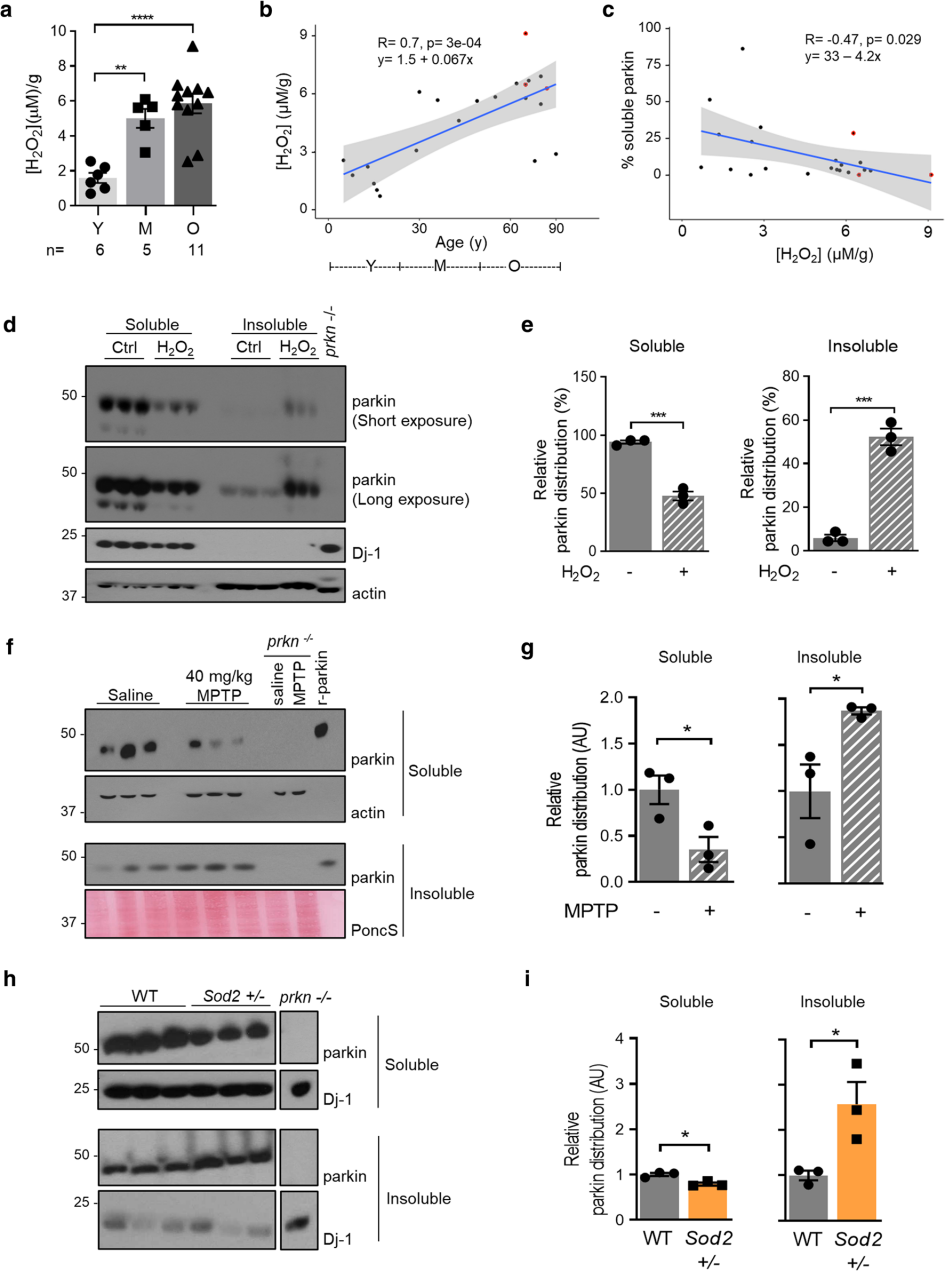
Decline in parkin solubility correlates with a rise of oxidative stress in mammalian brain. **a** Mean concentrations of H_2_O_2_ in human brain cortices grouped by age range, as described in Fig. 1. Individual data points represent separate brains, as reported in Supplementary Table 1, online resource. Results are plotted as mean ± SEM; significance was determined using 2-way ANOVA [*F*(2, 76) = 26.21, *p* < 0.001] with Tukey’s post hoc test (**p < 0.01; ***p < 0.001). **b–c** Linear regression analysis of H_2_O_2_ concentrations in control cortices (μM/g tissue) as a function of age **(b**), and **c** linear regression analysis of parkin solubility as a function of H_2_O_2_ levels in the same specimens (*n* = 22). Red circles denote three disease cortices (non-*PRKN*-linked parkinsonism). H_2_O_2_ concentration coefficient (in (**c**)) was − 4.2 (95% CI: − 7.92 to − 0.48, *P* = 0.0287). **d–e** Western blots **(d)** of parkin distribution in brain lysates of 2–4 month-old wild-type C57Bl/6 J mice containing either saline or 1% H_2_O_2_; **e** parkin signal distribution was quantified using ImageJ, as controlled for respective loading controls, in both soluble and insoluble fractions. A student t-test was used for statistical analysis (*p < 0.05). **f, g** Western blots **f** of parkin distribution in brains of wild-type C57Bl/6 J mice 1 h following intraperitoneal administration of either saline or MPTP neurotoxin (40 mg/kg); **g** parkin signals were quantified as in (**e**). **h–i** Western blots **h** of fractionated brain homogenates from C57Bl/6 J wild-type and *Sod2*^±^ mice; **i** parkin signals were quantified and statistically analyzed as in (**e**) (*p < 0.05). Note, Western blots shown in this figure followed SDS/PAGE under reducing conditions

We next explored a possible association between parkin distribution, age and oxidative changes. Using sister aliquots from the brain specimens examined above, we found that hydrogen peroxide (H_2_O_2_) concentrations positively correlated with age (Fig. 2a, b; see also Supplementary Table 1, online resource), as expected from the literature [54]. Using univariate linear regression analysis, we determined that the coefficient of age was 0.067 (95% CI: 0.035 to 0.098, *P* = 3e^−04^; multiple R-squared value, 0.4877).

In three brains from subjects with non*-PRKN*-linked parkinsonism, the levels of H_2_O_2_ were similar to those measured in age-matched controls (Fig. 2b). When analyzing parkin distribution vs*.* H_2_O_2_ concentrations in human cortices, we found that parkin solubility in human brain negatively correlated with H_2_O_2_, where the coefficient of the latter was − 4.2 (95% CI: − 7.92 to − 0.48, *P* = 0.029; multiple R-squared value, 0.2174) (Fig. 2c).

We next sought to dynamically model the observed correlation between higher ROS levels in the nervous system and reduced parkin solubility. We first used an ex vivo approach, whereby wild-type mouse brains were exposed to either saline or H_2_O_2_ during tissue homogenization. There, we saw a significant reduction in soluble parkin and an increase in insoluble parkin in the H_2_O_2_-exposed lysates (Fig. 2d, e). We next examined two in vivo models. In the first, wild-type mice were intraperitoneally injected with 40 mg/kg of 1-methyl-4-phenyl-1,2,3,6-tetrahydropyridine (MPTP) toxin one hour before sacrificing them to induce acute oxidative stress, but no cell death [3]. Brains were serially fractionated, and parkin distribution was quantified across soluble and insoluble compartments. There, we measured a decrease of murine parkin in the soluble fraction and a corresponding rise in the insoluble fractions of MPTP- *vs.* saline-injected littermates (Fig. 2f, g).

In the second in vivo model, we observed a similar, significant shift in parkin distribution toward insolubility in adult mice that were haploinsufficient for the *Sod2* gene, which encodes mitochondrial MnSOD, and which occurred in the absence of an exogenous toxin (Fig. 2h, i). Of note, in both models we confirmed the expected rise in H_2_O_2_ levels (see below and El-Kodsi et al*.* [17]). Moreover, in contrast to murine parkin, the solubility of endogenous Dj-1, which is encoded by a second, ARPD-linked gene*,* was not visibly altered under these elevated oxidative stress conditions, as monitored by SDS/PAGE/Western blotting (Fig. 2h).

### Parkin is reversibly oxidized in the adult human brain

**Fig. 3:**
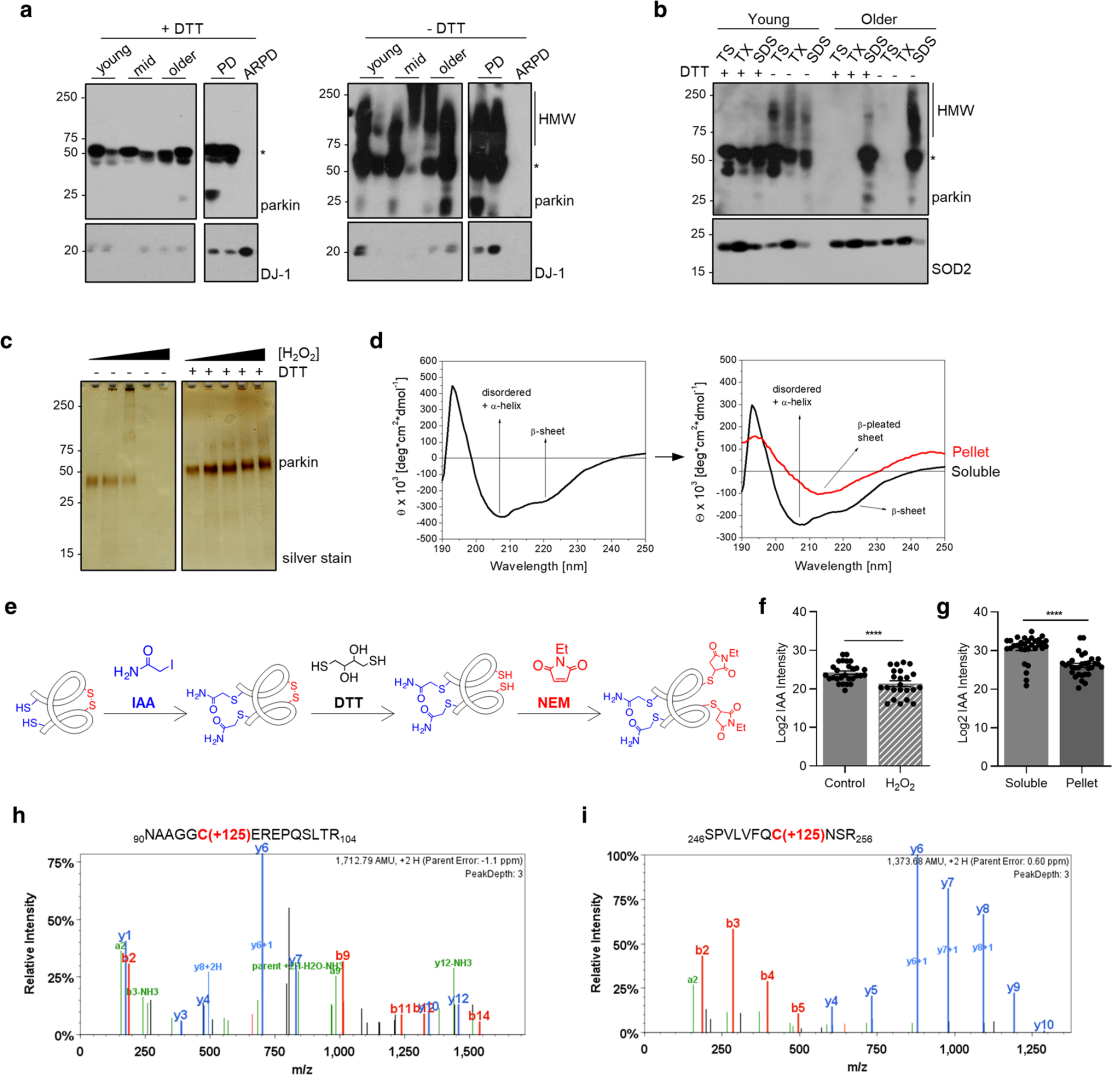
Parkin’s solubility and structure are altered by oxidative modifications. **a** Western blots of parkin and DJ-1 in SDS fractions from normal cortices (3 age groups are shown) and two age-matched patients, i.e., idiopathic Parkinson’s (PD) and parkin-deficient ARPD. Sister aliquots of the same lysates were processed in parallel by SDS-PAGE either under reducing (+DTT) or non-reducing (-DTT) conditions. **b** Western blots of parkin and SOD2 distribution in serially fractionated human cortices from a young individual (age, 5 years) and an adult (62 years) subject, and separated by SDS-PAGE under reducing (+DTT) and non-reducing (−DTT) conditions. **c** Silver staining of the supernatant of sister aliquots of r-parkin following initial exposure to increasing concentrations of H_2_O_2_ (0–2 mM) followed by the addition (or absence of) DTT (100 mM) prior to centrifugation as indicated. **d** Circular dichroism spectra of soluble, untreated, wild-type r-parkin at the start of experiment (*T* = 0; left panel), and spectra of soluble (black line) and aggregated (red line) states following incubation at 37 °C for *T* = 5 days (right panel). **e** Graphic depiction of strategy for LC–MS/MS-based analysis to identify cysteine oxidation state for untreated and H_2_O_2_-treated, parkin species, by using IAA-DTT-NEM fingerprinting to identify reduced cysteines with an iodacetamide (IAA) tag or reversibly-oxidized residues with a* N*-ethylmaleimide (NEM) tag. **f, g** Quantitative analyses of IAA-modified cysteines captured by LC–MS/MS for **f** untreated vs*.* H_2_O_2_-exposed, wild-type, human r-parkin, and **g** soluble compared to insoluble (pellet) fractions. Each dot represents the log2-transformed total IAA-signal intensities of individual cysteines (*n* = 3 runs for each). The cysteine pool is shown with the mean ± SEM; significance **p < 0.01, as determined using Student *T*-Test. **h–i** LC–MS/MS-generated spectra following trypsin digestion of labelled, oxidized r-parkin indicating NEM adducts (+ 125 mass gain) at Cys95 and Cys253; r-parkin was exposed to H_2_O_2_, and cysteines labelled as in (**e**). See Supplementary Table 2, online resource, for a complete list of modified cysteines and oxidizing conditions

The correlation between parkin insolubility and H_2_O_2_ levels in the human brain suggested to us that the relation could be due to posttranslational, oxidative modifications. Indeed, in contrast to SDS-containing brain fractions analyzed under reducing conditions (+ DTT), when gel electrophoresis was performed under non-reducing (-DTT) conditions, we detected parkin proteins ranging in *M*_*r*_ from > 52 to 270 kDa, invariably in the form of redox-sensitive, high molecular weight (HMW) smears (right vs*.* left panel; Fig. 3a). We saw the same pattern in fractions prepared from control midbrains; no such reactivity was seen in SDS-extracts of parkin*-*deficient ARPD brains, thus demonstrating the specificity of protein detection.

We confirmed that reversible oxidation of brain parkin was also present in soluble (TS-, TX-) fractions, albeit at lesser intensities (Fig. 3b; data not shown). Of note, the formation of high *M*_*r*_ parkin was not due to secondary oxidation in vitro*,* because specimens had been processed and fractionated in the presence of iodoacetamide (IAA) prior to SDS/PAGE in order to protect unmodified thiols. These HMW parkin smears also did not arise from covalent ubiquitin-conjugation, such as due to auto-ubiquitylation of parkin, owing to the fact that such adducts cannot be reversed by reducing agents (e.g*.,* DTT) (data not shown).

Because we predicted that the loss of parkin solubility was due to thiol-based, posttranslational oxidation events [50], we first sought to test this in vitro using purified, tag-less, full-length, recombinant (r-) parkin. There, we observed the H_2_O_2_ dose-dependent formation of HMW smears and loss of parkin solubility; however, r-parkin protein solubility was greatly recovered by adding DTT (Fig. 3c; Supplementary Fig. 3a, online resource) or β-mercaptoethanol (not shown). Demonstrating its sensitivity to bi-directional redox forces, the exposure of native r-parkin to excess DTT also rendered it increasingly insoluble (Supplementary Fig. 3b, online resource), likely due to loss of zinc-sulfur chelation in its four RING domains [31, 47]. Unlike r-parkin, the addition of up to 1 M DTT in the extraction buffer did not induce parkin’s extraction into a soluble phase (i.e*.,* TS- or TX-fractions) in aged human brain tissue (Supplementary Fig. 3c, online resource).

We confirmed by mass spectrometry (MS) of the holoprotein carried out without any trypsin digestion that all 35 cysteine-based thiol groups of human r-parkin are principally accessible to alkylation by IAA (right *vs.* left panel; Supplementary Fig. 3d, online resource). These results unequivocally demonstrated that each parkin cysteine theoretically possesses the capacity to have its thiol be modified. Nevertheless, in these in vitro experiments we consistently observed a concentration-dependent change in r-parkin solubility, thereby suggesting that some thiols were more amenable than others to modification by reactive species (see below and summary in Supplementary Table 2, online resource).

### Oxidative conditions alter parkin structure

The progressive insolubility of brain parkin and r-parkin due to redox stress suggested that the protein had undergone structural changes. Indeed, when we analyzed the effects of spontaneous oxidation using native r-parkin by far-UV-circular dichroism (Fig. 3d), soluble fractions initially contained both α-helically ordered as well as unstructured r-parkin proteins. Five days later, r-parkin preparations were separated by centrifugation and fractions re-analyzed. There, we found a marked shift to increased β-pleated sheet-positive r-parkin in insoluble fractions (Fig. 3d). Similarly, when we monitored r-parkin during spontaneous oxidization using dynamic-light scattering (Supplementary Fig. 3e, online resource), we observed a gradual shift in the hydrodynamic diameter from 5.1 nm, representing a folded monomer, to multiple peaks with larger diameters 5 h later. The latter indicated spontaneous multimer formation, which was partially reversed by the addition of DTT (right panel; Supplementary Fig. 3e, online resource). Thus, these structural and solubility changes of r-parkin were congruent with our immunoblot results for human brain parkin (Fig. 3a).

### Hydrogen peroxide modifies parkin at multiple cysteines

To determine whether the oxidation of cysteines and/or methionine residues caused parkin insolubility, we analysed r-parkin that was treated with and without H_2_O_2_ and/or thiol-alkylating agents using liquid chromatography-based MS (LC–MS/MS). To differentiate reduced from oxidized cysteines we used a serial thiol-fingerprinting approach, which labelled reduced thiols with IAA, and tagged reversibly oxidized thiols with *N*-ethylmaleimide (NEM) after their prior reduction with DTT (Fig. 3e). The first test was to determine how progressive oxidation affected thiol accessibility. As expected, using the strong alkylating agent IAA on the nascent protein (and trypsin digestion to map individually modified peptides), we confirmed that the majority of parkin cysteines were reactive (Supplementary Fig. 3d; Supplementary Table 2, online resource). Intriguingly, when treating native r-parkin with lower H_2_O_2_ concentrations, we identified an average of 19 cysteines (54.3%) to be modified; in contrast, higher H_2_O_2_ concentrations increased this number to 32 cysteines (91.4%). These results suggested progressive unfolding of r-parkin with increasing oxidation (Supplementary Table 2, online resource).

Next, we sought to precisely identify the location of oxidized cysteine residues. Using Scaffold PTM-software, we found a rise in the number of oxidized residues (NEM-Cys, range of 3–26), which was proportional to the increase in H_2_O_2_ concentrations and appeared to begin in parkin’s RING1 domain at three residues, i.e*.,* Cys238, Cys241 and Cys253 (Supplementary Table 2, online resource; Fig. 3i), but also involved Cys95 in its linker domain (Fig. 3h). Furthermore, when quantifying thiol modifications by MaxQuant software [10], we found a significant drop for the number of cysteines in the reduced state (IAA-cysteines) within the H_2_O_2_-treated samples (*P* = 0.0016; Fig. 3f), as expected.

In accordance, when comparing cysteine oxidation events in soluble and insoluble fractions of untreated *vs.* oxidized r-parkin preparations, the number of IAA-Cys was significantly decreased in the pellets (*P* < 0.0001; Fig. 3g). Of note, modifications at methionine residues did not correlate with r-parkin solubility. These collective results unequivocally demonstrated that H_2_O_2_-induced oxidation events at cysteine-based thiols are linked to both progressive, structural change and lesser solubility of human r-parkin.

### Parkin is also irreversibly oxidized in adult human and mouse brains

**Fig. 4:**
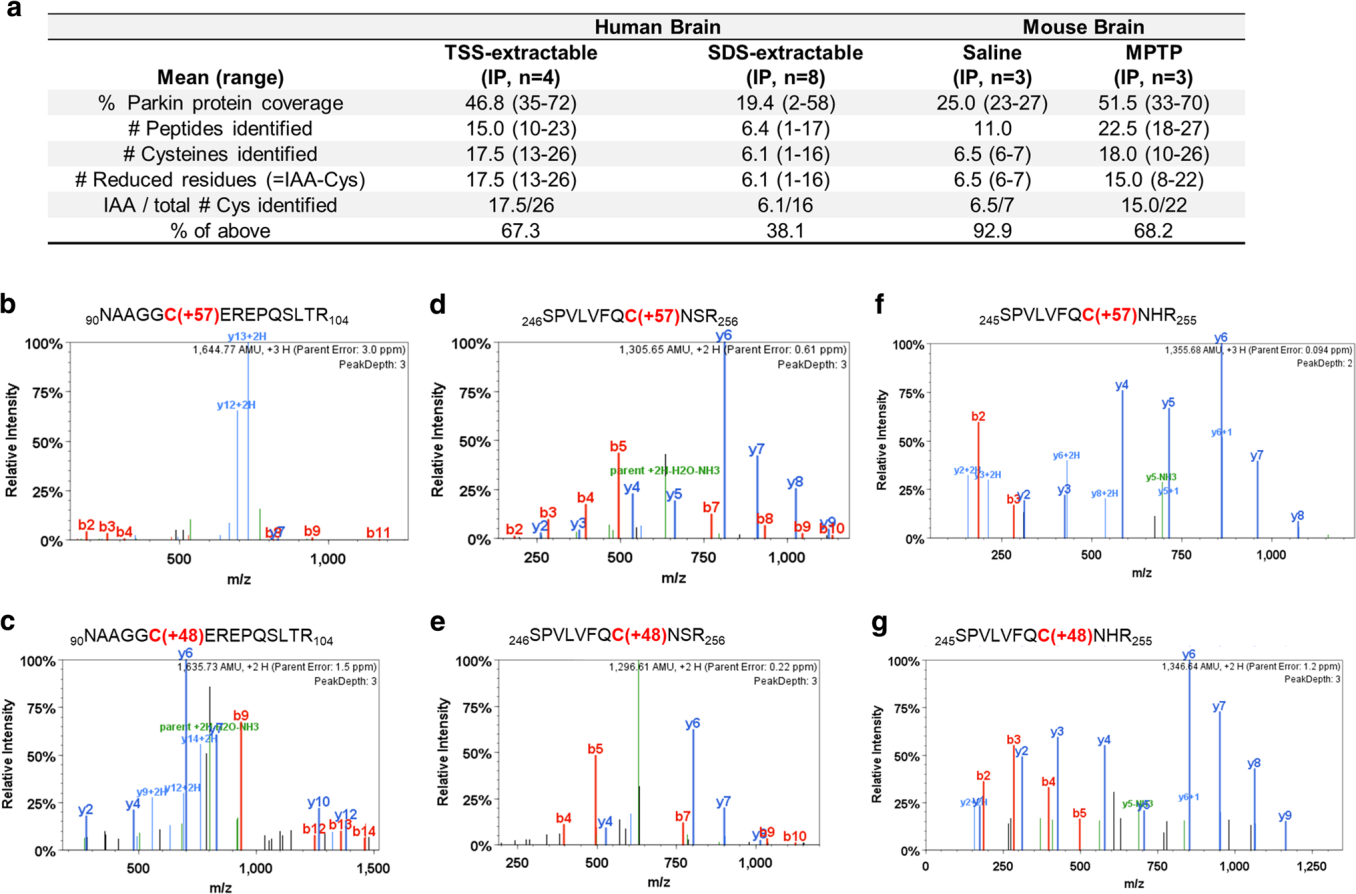
Select parkin cysteine residues are oxidized in human and mouse brain. **a** Summary of results for 12 immunoprecipitation (IP) runs (TS extracts; *n* = 4; SDS extracts, *n* = 8) from human cortices and either saline- or acute (1 h) MPTP toxin-treated murine brain (as described in Fig. 2d, e) for endogenous parkin enrichment to identify the redox state of its cysteine residues (see also **b**–**g**). All specimens were fractionated in the presence of IAA. **b–g** Among the redox active residues identified Cys95 and Cys253 in human brain parkin were found in either a reduced redox state **(b, d)** (i.e., IAA-labelled; + 57 mass gain) or **(c, e)** in irreversibly oxidized states, e.g., to sulfonic acid (trioxidation; + 48 mass). In mouse brain parkin **(f, g)**, Cys252 was found either reduced or oxidized as well

We next sought to identify oxidation events at parkin cysteines in vivo by LC–MS/MS. To this end, we examined both cortex-derived, human parkin and brain parkin isolated from intraperitoneally, MPTP toxin- (vs*.* saline-) treated mice (Fig. 4). Specimens were processed with IAA during homogenization and fractionation to prevent any oxidation artefacts in vitro. Following immunoprecipitation and gel excision of endogenous parkin at the 50–53 kDa range (an example is shown in Supplementary Fig. 4a, b, online resource), we focused on cysteine mapping and the identification of thiol redox states (Fig. 4a). A graphic representation of theoretically possible, thiol-based redox modifications is provided in Supplementary Fig. 4c, online resource).

In human control cortices (*n* = 12 runs; summarized in Fig. 4a), we mapped a mean of 46.8 and 19.4% of parkin’s wild-type sequence in the soluble and insoluble fractions, respectively. There, we found cysteines in either a redox reduced state (IAA-alkylated Cys + 57; examples shown in Fig. 4b, d) or in oxidized states (e.g., to sulfonic acid Cys + 48). Irreversible oxidation events in human cortices occurred, for example, at Cys95 (Fig. 4c) and Cys253 (Fig. 4e). The relative frequencies of detection for parkin thiols that were found in a reduced state in vivo (and thus, were alkylated by IAA in vitro) in the soluble *vs.* insoluble fractions of the human brain were 67.3 vs*.* 38.1%, respectively (Fig. 4a).

Likewise, in saline- and MPTP-treated mouse brains (*n* = 6 runs), we mapped 25.0 and 51.5% of wild-type parkin, respectively (summarized in Fig. 4a). Interestingly, akin to the findings in the human brain, we identified the murine sequence-corresponding residue Cys252 in either a reduced or in irreversibly oxidized states (Fig. 4f, g). As mentioned, mice do not carry a cysteine at residue 95 (for sequence comparison, see below). The relative frequencies of detection for thiols that were in a reduced state in vivo (and thus, alkylated by IAA in vitro) in parkin from saline- vs*.* MPTP toxin-treated mouse brains were 92.9 vs. 68.2%, respectively (Fig. 4a). These collective results demonstrate that parkin cysteines are variably oxidized in adult mammalian brain.

### Parkin thiols reduce hydrogen peroxide in vitro

**Fig. 5:**
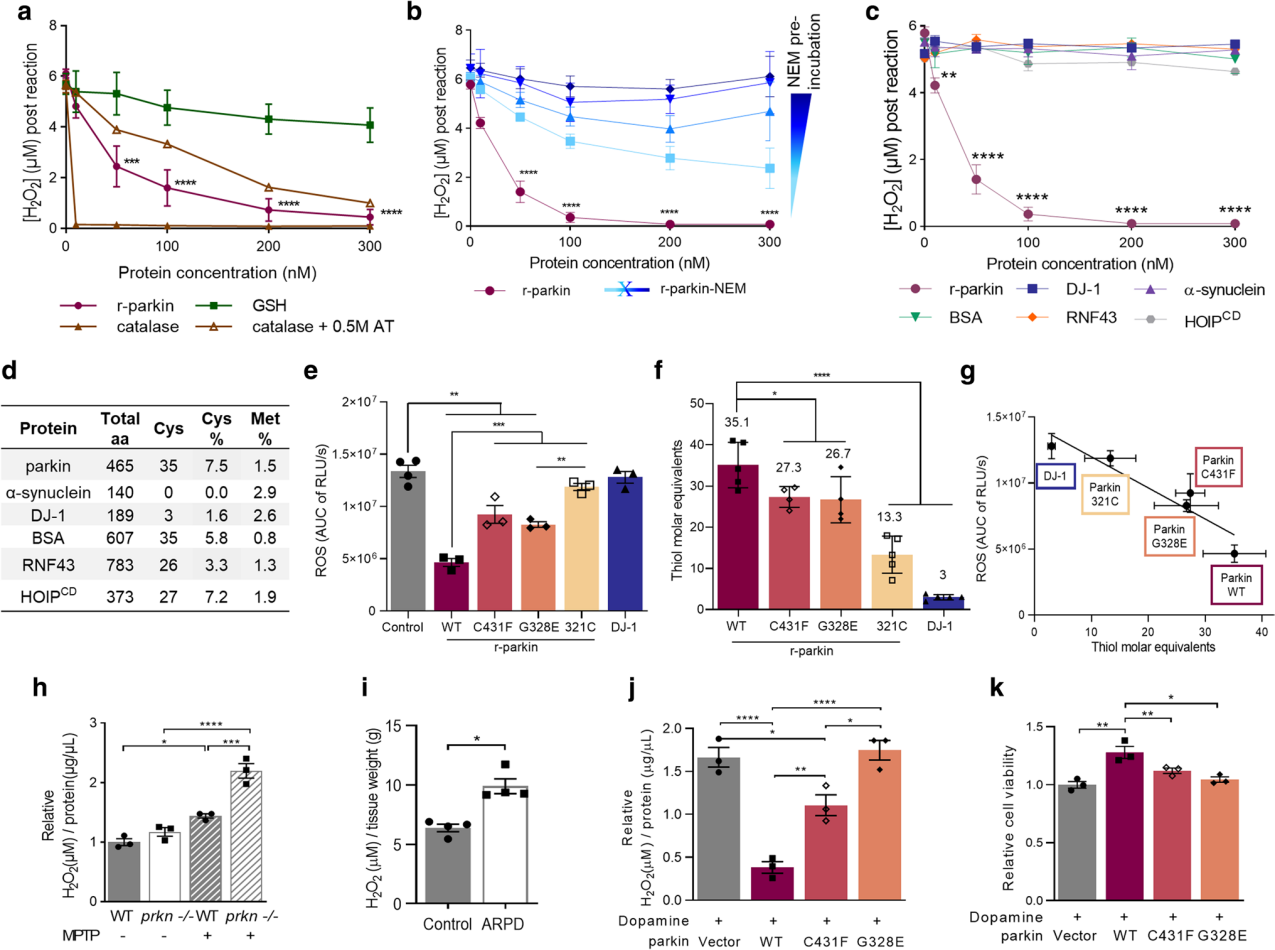
Wild-type parkin lowers hydrogen peroxide in vitro, in cells and the brain. **a–c** Quantification of H_2_O_2_ concentration using AmplexRed, demonstrating **(a)** full-length, human, recombinant (r-) parkin when incubated with H_2_O_2_ is able to reduce it in a r-parkin concentration-dependent manner. Effects of r-Parkin were compared to catalase and GSH at equimolar concentrations as well as following partial inhibition of catalase by amino-triazole (AT), as indicated. **b** Pre-incubation of r-parkin with a thiol-conjugating compound (NEM) inhibits parkin-dependent H_2_O_2_ reduction in a NEM-concentration-dependent manner. **c** Reducing capacity of wild-type r-parkin compared to two other, PD-linked proteins (DJ-1; α-synuclein), bovine serum albumin (BSA) and two RING-carrying ubiquitin ligases (RNF43; HOIP^cd^; cd = catalytic domain). Their respective cysteine and methionine contents are summarized in **(d)**. Two-way ANOVA with Tukey’s post hoc test (**p < 0.01, ***p < 0.001, and ****p < 0.0001) was used for statistical analysis **a** [*F* (15, 48) = 5.069, *p* < 0.0001]; **b** [*F* (20, 60) = 3.966, *p* < 0.0001]; and **c** [*F* (25, 72) = 22.91, *p* < 0.0001]. **e** Area under the curve (AUC) plots for results from in vitro colorimetric assays, where AUC integrates total H_2_O_2_ levels measured over the time course of the assay (see also Supplementary Fig. 5f, online resource). Comparison of WT r-parkin with DJ-1, two r-parkin point mutants, and r-parkin_321-465_ (321C). Results represent *n* = 3 ± SD using one-way ANOVA [*F* (7, 17) = 99.87, *p* < 0.0001] with Tukey’s post hoc test *p < 0.05, **p < 0.01,***p < 0.001, and ****p < 0.0001. **f** Quantification of reactive thiol content (in molar equivalents) for r-parkin (WT; two point mutants; 321C) and full-length r-DJ-1 using the Ellman’s reagent assay. Results analyzed by one-way ANOVA [*F* (4, 18) = 45.11,* p* < 0.0001]. **g** Correlation curve between number of free thiols **(f)** vs. the H_2_O_2_-reducing capacity **(e)** for indicated proteins with *R*^2^ = 0.8789. **h–i** Quantification of H_2_O_2_ levels in **(h)** saline vs*.* MPTP toxin-treated *prkn* wild-type (WT) and *prkn*^−/−^ mouse brain (*n* = 3/genotype/condition), and **i** in human brain from parkin-deficient ARPD cortices compared to age- and *post-mortem* interval-matched controls (*n* = 4/group) collected at the same institution. Results are represented as the mean concentration of H_2_O_2_ (μM) per total protein concentration (μg/μL) or tissue weight (**g**) analyzed ± SEM; *p < 0.05, ***p < 0.001, and ****p < 0.0001 determined using a Student *T*-test or one-way ANOVA with Tukey’s post hoc test; [*F* (3, 8) = 45.41, *p* < 0.0001]. **j, k** H_2_O_2_ quantification **(j)** and cell viability assay **(k)** for dopamine-treated, human M17 cells expressing either WT or two ARPD-linked parkin point mutants, as indicated relative to treatment with vehicle alone. Cells were exposed to 200 μM dopamine or vehicle for 20 h, as indicated. Data points represent the mean of duplicates ± SEM (*n* = 3 experiments); *p < 0.05 and **p < 0.01, and ****p < 0.0001 by one-way ANOVA with Tukey’s post hoc test: **j** [*F* (3, 8) = 35.34, *p* < 0.0001]; and **k** [*F* (3, 8) = 12.92, *p* = 0.0020]

A typical redox reaction involves the reduction of an oxidized molecule in exchange for the oxidation of the reducing agent (examples are shown in Supplementary Fig. 4c, online resource). We, therefore, asked whether parkin oxidation resulted in a reciprocal reduction of its environment (Fig. 5; Supplementary Fig. 5, online resource). Using r-parkin, we established that parkin could reduce H_2_O_2_ levels in a concentration-dependent manner in vitro (Fig. 5a; Supplementary Fig. 5h, online resource). This reducing activity was not enzymatic, in that it did not mirror the dynamics of catalase, and r-parkin did not possess peroxidase activity (Fig. 5a; Supplementary Fig. 5a, online resource). Rather, the reaction was dependent on parkin’s thiol integrity, because pre-treatment with NEM (or IAA) and pre-oxidation of the protein with H_2_O_2_ abrogated the ROS-reducing activity of r-parkin (Fig. 5b; Supplementary Fig. 5b, g, online resource). It thus appeared similar to the effect of glutathione (Fig. 5a; Supplementary Fig. 5a, e, f, online resource).

The anti-oxidant effect by r-parkin was also dependent on its intact Zn^2+^ coordination, because increasing concentrations of the divalent ion chelator, EDTA, abrogated the activity; the latter could be ameliorated by supplementing the reaction buffer with zinc (Supplementary Fig. 5c, online resource). As expected, the exposure of r-parkin to excess H_2_O_2_ (or excess DTT) led to the release of zinc ions from the nascent recombinant protein, as measured in vitro (Supplementary Fig. 5d, online resource).

Interestingly, RNF43 (a distinct E3 ligase that contains a zinc-finger domain), HOIP (an E3 ligase containing a RING domain) and bovine serum albumin (BSA, which akin to parkin has 35 cysteines), did not show any H_2_O_2_-lowering capacity (Fig. 5c, d; Supplementary Fig. 5e, online resource). Further, Parkinson’s-linked α-synuclein, which has no cysteines, also had no reducing effect (Fig. 5c, d). These results suggested that the cysteine-rich, primary sequence and the tertiary structure of r-parkin conferred anti-oxidant activity.

We next examined an additional, cysteine-containing, ARPD-linked protein, *e.g.*, r-DJ-1 and two disease-linked variants of full-length r-parkin, p.G328E and p.C431F, as well as a C-terminal RING2-peptide of parkin (r-parkin_321C_). We also used a second ROS quantification assay for further validation and to expand our dose-dependency studies (Fig. 5e, Supplementary Fig. 5f–m, online resource). There, r-DJ-1 and r-parkin_321C_ showed negligible H_2_O_2_-lowering capacity, and the two point-mutants conferred less activity than did wild-type, human r-parkin (Fig. 5e). As expected from typical redox reactions (Supplementary Fig. 4c, online resource), the lowering of ROS in vitro correlated with reciprocal r-parkin oxidation, as revealed by SDS/PAGE, which was performed under non-reducing conditions immediately after the reaction with H_2_O_2_ (Supplementary Fig. 5n, online resource).

These results suggested that the anti-oxidant activity by r-parkin was dependent on its reactive thiol content, which we examined next using the Ellman’s reagent. There, full-length r-parkin, r-parkin_321C_ and r-DJ-1 showed the predicted number of reactive thiols, whereas the single point-mutant variants of r-parkin revealed fewer accessible thiols (Fig. 5f). From these results, we observed a linear correlation between thiol equivalencies and the degree of ROS reduction in vitro, demonstrating that a greater number of readily reactive and/or a greater number of accessible thiols in human parkin proteins corresponded with a more effective lowering of H_2_O_2_ (Fig. 5g).

### Hydrogen peroxide levels are increased in parkin-deficient brain

To explore whether parkin oxidation conferred ROS reduction in vivo, we first quantified H_2_O_2_ concentrations in the brains of wild-type and *prkn*^−/−^ mice. A trend, but no significant difference, was measured under normal redox equilibrium conditions. However, when analyzing brain homogenates from mice treated with MPTP-toxin *vs.* saline, carried out as above (Fig. 2), we found significantly higher H_2_O_2_ levels in the brains of adult *prkn*^−/−^ mice compared to wild-type littermates (*P* < 0.001; Fig. 5h). Similarly, in adult humans H_2_O_2_ levels were significantly increased in the cortex of *PRKN-*linked ARPD patients *vs.* age-, PMI-, ethnicity- and brain region-matched controls [42] (*P* < 0.05; Fig. 5i). Specimens of three non*-PRKN*-linked patients with parkinsonism showed H_2_O_2_ levels comparable to those from age-matched normal cortices (Fig. 2b, red circles). We concluded that the expression of wild-type *PRKN* alleles contributes to the lowering of ROS concentrations in adult, mammalian brain.

### Parkin prevents dopamine toxicity in cells in part by lowering hydrogen peroxide

To address the question of selective neuroprotection, we revisited the role of parkin in cellular dopamine toxicity studies [51, 104]. We first tested parkin’s effect on ROS concentrations in dopamine-synthesizing, human M17 neuroblastoma cells. There, dopamine exposure of up to 24 h caused a significant rise in endogenous H_2_O_2_ (*P* < 0.05; Fig. 5j), as expected. Wild-type *PRKN* cDNA expression effectively protected M17 cells against the dopamine stress-related rise in H_2_O_2_ levels (*P* < 0.0001; Fig. 5j). By comparing sister cultures that expressed similar amounts of exogenous parkin proteins, the E3 ligase-inactive p.C431F mutant had a partial rescue effect, whereas p.G328E, which we confirmed to retain its E3 ligase activity in vitro, showed no H_2_O_2_-lowering capacity in these cells (Fig. 5j; and data not shown).

Moreover, only wild-type parkin, but none of the mutant variants tested, increased the viability of M17 cells under rising dopamine stress conditions (*P* < 0.01; Fig. 5k; and data not shown). This protective effect also correlated with parkin insolubility and its HMW smear formation, as expected from previous studies [51]. These posttranslational changes in M17-expressed parkin were not reversible by DTT or SDS (Supplementary Fig. 6a, b, online resource), thereby suggesting irreversible dopamine-adduct formation. Notably, the protection from dopamine toxicity positively correlated with the level of *PRKN* cDNA transcribed, as confirmed in sister lines of M17 cells that stably express human, wild-type parkin. There, we estimated that ~ 4 ng of parkin protein expressed in healthy, neural cultures neutralized each μM of dopamine added during up to 24 h (Supplementary Fig. 6c, d, online resource).

### Parkin binds dopamine radicals predominantly at primate-specific cysteine 95

**Fig. 6:**
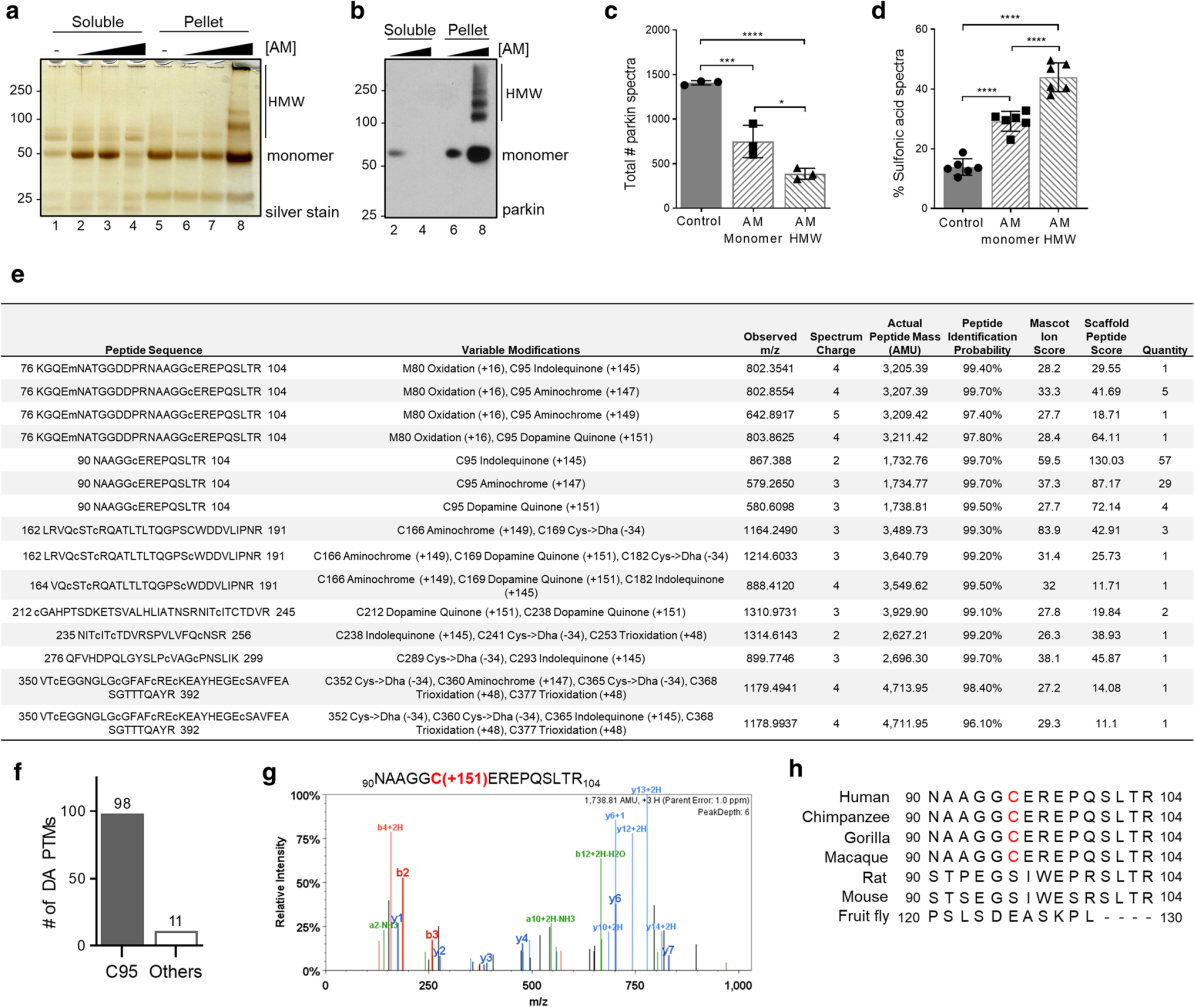
Human parkin conjugates dopamine radicals foremost at residue Cys95. **a, b** Silver staining **(a)** and Western blot **(b)** of r-parkin in soluble (supernatant) and insoluble (pellet) phases following exposure to increasing concentrations of aminochrome (AM; 0–200 μM) and analyzed under non-reducing conditions. See lane number for corresponding samples. **c** Mean total number of parkin spectra, as identified by LC–MS/MS following trypsin digestion, of control *vs.* monomeric *vs.* high molecular weight (HMW), AM-modified r-parkin. Data represent the mean of *n* = 3 runs ± SEM. *p < 0.05; ***p < 0.001; ****p < 0.0001 by one-way ANOVA with Tukey’s post-hoc test [*F* (2,6) = 64.73, *p* < 0.0001]. **d** Percentage of peptides carrying a sulfonic acid modification in control vs*.* monomeric and HMW, AM-modified r-parkin. Each point represents one gel specimen submitted to MS. The percentage was calculated using only the subset of peptides that were ever detected as carrying a sulfonic acid modification. Statistics were done as in (**c**) [**F** (2,15) = 96.87, *p* < 0.0001]. **e** Table summarizing LC–MS/MS-based detection of adducts representing dopamine metabolites conjugated to cysteines identified in human r-parkin following exposure to aminochrome in vitro. Chemical structures for identified cysteine-conjugated adducts are shown in Supplementary Fig. 7b, online resource. Individual quantification of each peptide with adduct listed is shown on the right side of the table. **f** Frequency of occurrences for dopamine-metabolite adducts being detected on Cys95 vs*.* all other cysteine residues, as detected by LC–MS/MS and individually shown in (**e**). **g** LC–MS/MS-generated spectrum following trypsin digestion of AM-exposed r-parkin highlighting a dopamine (+ 151 mass gain) adduct covalently bound to Cys95. See also Supplementary Fig. 7c–p, online resource, for additional spectra. **h** Species comparison for wild-type parkin proteins covering sequence alignment of aa90-104, with primate-specific residue Cys95 highlighted in red

We next explored which thiols of parkin were involved in the neutralization of dopamine radicals. Covalent conjugation of RES metabolites at parkin residues had been previously suggested [51, 104], but not yet mapped by LC–MS/MS examining the whole protein. Aliquots of r-parkin were exposed to increasing levels of the relatively stable dopamine metabolite aminochrome*.* As expected, this led to the loss of protein solubility and HMW species formation at the highest dose tested (Fig. 6a, b). These reaction products were then used to map modified residues by LC–MS/MS. Specifically, proteins corresponding to r-parkin monomer (51–53 kDa) and two HMW bands, one at ~ 100 kDa, the other near the loading well, were gel-excised (Fig. 6a), trypsin digested and analyzed.

There, we made the following four related observations: (i) Increasing aminochrome concentrations led to a significant decline in the total number of spectra readily identified by LC–MS/MS as parkin-derived peptides, both in the monomeric and HMW bands (*P* < 0.001 and *P* < 0.0001), respectively (Fig. 6c). This indicated to us either a marked loss in solubility (and thus, lesser accessibility by trypsin) or a rise in heterogenous, complex modifications, which rendered the analyte undetectable by LC–MS/MS, or both; (ii) Despite fewer spectra recorded, we identified a significant increase in the number of oxidized cysteines (such as irreversibly modified to sulfonic acid) following aminochrome exposure, in particular within the HMW bands of r-parkin (*P* < 0.0001; Fig. 6d); (iii) Under these conditions, four distinct forms of dopamine metabolites were found conjugated to parkin cysteines. Mass shifts of + 145, + 147, + 149 and + 151 were identified, which represented covalent attachment by indole-5,6-quinone, two variants of aminochrome (O = ; HO–), and dopamine quinone itself, respectively (Fig. 6e; Supplementary Fig. 7a, online resource); and (iv) Unexpectedly, we identified in Cys95 the most frequently dopamine-conjugated parkin residue (*P* < 0.0001; *n* = 98 spectra; Fig. 6e–g; Supplementary Fig. 7b–g, online resource). Other residues of r-parkin, which we identified to carry any one of the dopamine metabolites we tracked, included Cys166, Cys169, Cys182, Cys212, Cys238, Cys293, Cys360 and Cys365, but at a much lesser frequency (Fig. 6e, f; Supplementary Fig. 7h–o, online resource). No dopamine metabolite-related mass shifts were detected in the control samples that had not been exposed to aminochrome, as expected. We noted with interest that residue Cys95 of wild-type parkin, as the most frequently catalogued one to be modified by dopamine metabolites, is also primate sequence-specific (Fig. 6g, h).

### Parkin augments melanin formation in vitro, which involves residue cysteine 95

**Fig. 7:**
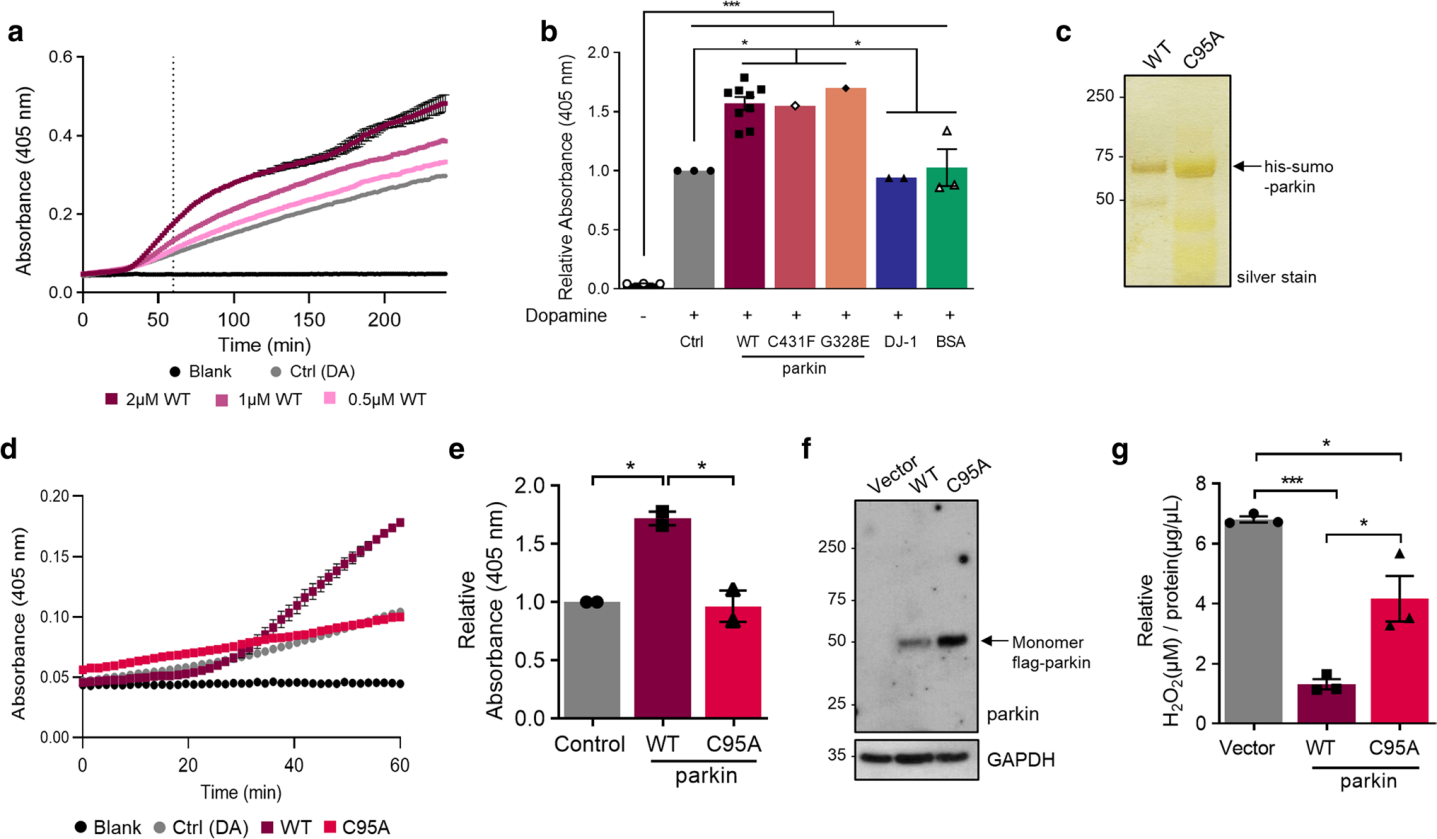
Parkin-dependent increase in melanin formation involves residue cysteine 95. **a** Kinetic curve of melanin production (read at absorbance 405 nm) over time in the absence of exogenous protein (dopamine (DA Ctrl) alone) *vs.* increasing molar concentrations of wild-type (WT), full-length human r-parkin shown for three concentrations (0.5, 1, 2 μm). Each condition was performed in triplicate. **b** Total melanin formation for indicated recombinant proteins at 60 min, as expressed relative to its production under dopamine only control (Ctrl) condition. Data represent the mean of triplicates ± SEM. ***p < 0.05 by 1-way ANOVA with Tukey’s post-hoc test [*F*(6,15) = 40.05, *p* < 0.0001]. **c** Silver gel for the analysis of His-SUMO-tagged, full-length, human r-parkin proteins of wild-type sequence and its variant carrying a p.C95A mutation. SDS/PAGE was performed under reducing conditions. **d, e** Representative kinetic curve for melanin production **(d)** and relative total melanin formation at 60 min **(e)**, where production in the presence of wild-type (WT) or p.C95A mutant r-parkin (each, 2 μM) is shown relative to dopamine (DA) (Ctrl) alone. Data represent mean of *n* = 2, each performed in triplicate ± SEM. ***p < 0.05 by 1-way ANOVA with Tukey’s post-hoc test [*F*(2,3) = 24.96, *p* = 0.0135]. **f, g** Protein expression, as shown by Western blotting **(f)**, and fold change in H_2_O_2_ levels **(g)** for dopamine-treated M17 cells -relative to vehicle-treated sister wells- that transiently express either flag-control, or WT vs*.* p.C95A-mutant human parkin-encoding cDNA plasmids. Results are shown as mean ± SEM (*n* = 3) and all dopamine-treated samples (200 μm dopamine) were normalized to their respective untreated samples. Anti-GAPDH immunoblotting served as a loading control (in **f**). A one-way ANOVA with Tukey’s post hoc test (*p < 0.05 and ***p < 0.001) was used for statistical analysis; [*F* (2, 6) = 36.86, *p* = 0.0004]

The oxidation of dopamine in the presence of cysteine-containing proteins, which generates covalent adduct-carrying proteins, underlies structural characteristics during the formation of neuromelanin pigment in the human midbrain (and pons), of which biochemical aspects have been modeled ex vivo [18, 19]. Given the observed relations between r-parkin, dopamine radical conjugation, aggregate formation and protein insolubility, we next examined whether melanin formation was altered by the presence of parkin. Indeed, wild-type r-parkin augmented total melanin formation in a protein concentration- and time-dependent manner in vitro (Fig. 7a). Like the wild-type protein, two ARPD-linked, full-length r-parkin variants, p.C431F and p.G328E, also augmented melanin formation in vitro, when monitored over 60 min, whereas r-DJ-1 and BSA showed no effect under these conditions (Fig. 7b).

Interestingly, mutagenesis of residue Cys95 to alanine (p.C95A; Fig. 7c), which was confirmed by nucleotide- and protein sequencing (by LC–MS/MS), completely abrogated the enhancing effect by r-parkin on the polymerization rate of dopamine to melanin (Fig. 7d, e). Of note, in our study all the recombinant proteins heretofore analyzed were used after their N-terminal His-SUMO-tag had been removed; however, the p.C95A-mutant was resistant to enzymatic digestion of the tag from the parkin holoprotein. Therefore, both His-SUMO-r-parkin and His-SUMO-p.C95A were utilized (Fig. 7c–e). Importantly, in parallel experiments we saw no difference in the kinetics of melanin formation between wild-type r-parkin proteins that either carried a His-SUMO*-*tag or were tag-less (not shown). We concluded that under these in vitro conditions, residue Cys95 was highly relevant to enhanced melanin polymerization by human parkin.

Furthermore, when the p.C95A-variant of parkin was expressed in M17 cells and examined in our dopamine toxicity assay, the mutant protein showed only a partial effect in H_2_O_2_ lowering capacity when compared to wild-type parkin, even when p.C95A was expressed at much higher levels (Fig. 7f, g). These results were consistent with our collective LC–MS/MS results of oxidative modifications of parkin at Cys95 (shown in: Figs. 3h, 4c; Supplementary Table 2, online resource). We reasoned from these complementary ex vivo results that wild-type parkin could be associated with the synthesis of neuromelanin in vivo. Therefore, we sought to explore this further in dopamine neurons of human midbrain.

### Anti-parkin reactivity localizes to neuromelanin in the *Substantia nigra* of adult control brain

Subcellular localization studies of parkin in human brains had previously been hindered by the lack of renewable antibodies (Abs) that reliably detect the protein in situ [73, 77, 81, 85]. We, therefore, developed and extensively characterized several, monoclonal Abs of the IgG_2_b-subtype using preparations of untagged, full-length, human r-parkin as immunogen*.* To this end, we generated four stable, epitope-mapped clones, i.e*.,* A15165B, A15165D, A15165G, and A15165E. The performance and specificity of these clones had been confirmed by ELISA, dot blot analyses, SDS/PAGE/Western blotting under reducing conditions, which included the usage of ARPD brain extracts, immunoprecipitation from the human brain and indirect immunofluorescence in cellular studies (Supplementary Fig. 8a–c, online resource; Tokarew et al., manuscript in preparation). Importantly, clones A15165D, A15165G, and A15165E were able to specifically detect human parkin in human brain sections by immunohistological methods (see below).

**Fig. 8:**
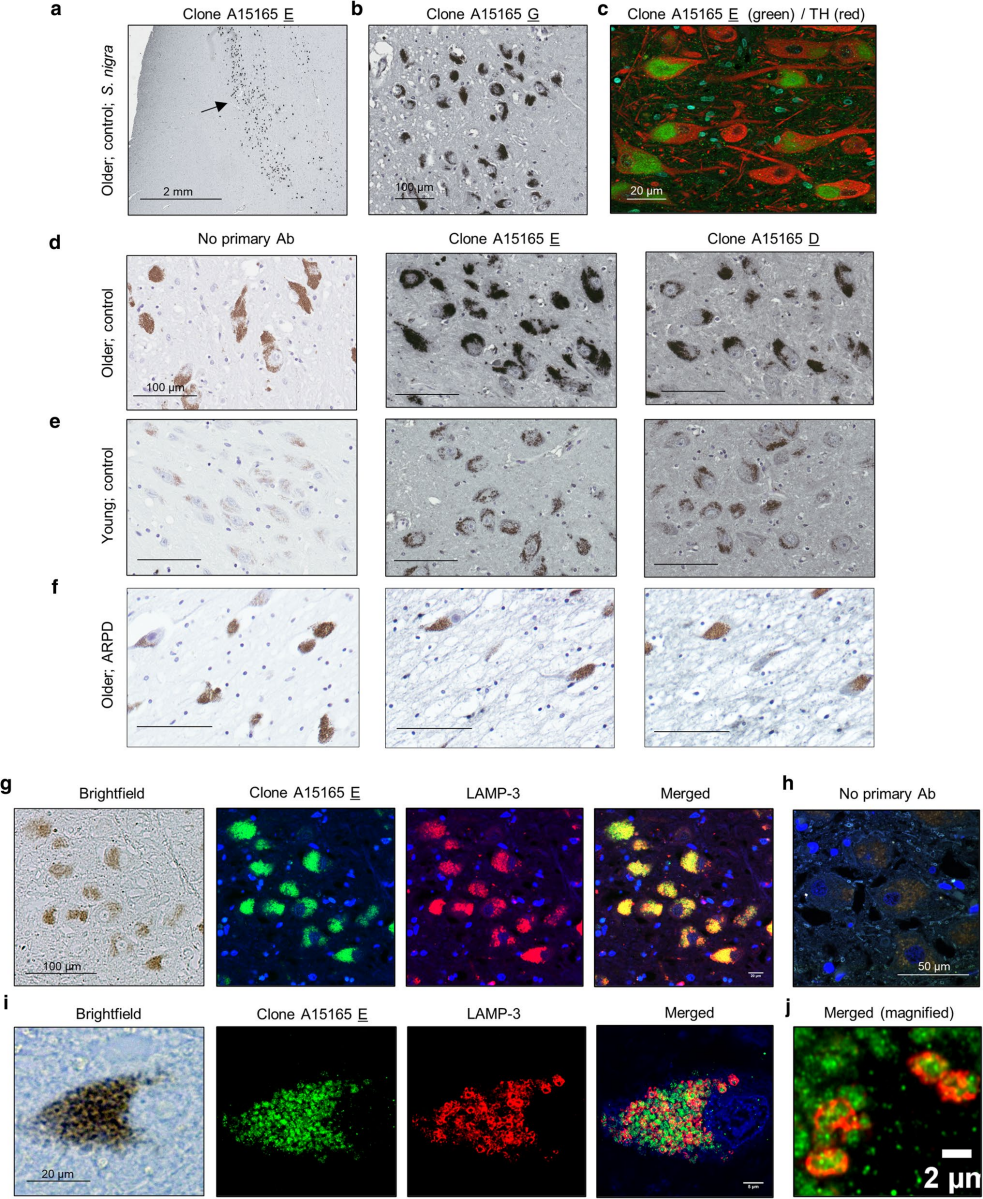
Parkin localizes to neuromelanin pigment in *S. nigra* neurons of normal human midbrain. **a, b** Immunohistochemical detection of parkin in the adult human brain including dopamine neurons of the *S. nigra* using anti-parkin monoclonal antibody clones A15165E **(a)** and -G **(b)**. **c** Double labelling for tyrosine hydroxylase (TH) and parkin (clone A15165E) in the *S. nigra* from an adult control subject using indirect immunofluorescence microscopy. **d–f** Immunohistochemical reactivities generated by no primary antibody vs*.* two anti-parkin (Clones A15165E, -D) antibodies on sections of the *S. nigra* from two control subjects, aged **(d)** 66 years and **(e)** 24 years, as well as **(f)** from a parkin-deficient ARPD case, aged 71 years. In the indicated panels, immunoreactivity was detected by metal-enhanced DAB (eDAB; generating black colour) and hematoxyline as a counterstain (blue). No primary antibody added generates a pigment-induced signal for neuromelanin (brown). Scale bars represent 100 μm, or as indicated. **g–j** Immunofluorescent signals, as generated by double-labelling of human *S. nigra* sections containing dopamine neurons, using anti-parkin (clone A15165E; green colour) and anti-LAMP-3/CD63 (red colour) antibodies; (blue colour, Hoechst stain). Brightfield microscopy image in the same field (neuromelanin pigment is visible; left panel) and a no primary antibody **(h)** run in parallel are shown. **i** Higher magnification of a single dopamine neuron and **(j)** further magnification for visualization of subcellular signals within a neighbouring dopamine neuron is shown, as indicated

Serial sections of adult, human midbrain from control subjects were developed by traditional immunohistochemistry (IHC) using metal-enhanced 3–3′-diaminobenzadine (eDAB), which generates a black signal for positive immunoreactivity. There, anti-parkin clones A15165D, A15165G and A15165E revealed dark, granular staining throughout the cytoplasm of pigmented cells (ages, ≥ 55 years) (Fig. 8a, b, d). Using sections of anterior midbrains from nine adult control subjects, ≥ 83% of the anti-tyrosine hydroxylase (TH)-positive neurons were also positive for parkin, as quantified by double labelling (Fig. 8c). Under these conditions and Ab concentrations, no anti-parkin signal was generated by clone A15165B, which had been successfully used in IP experiments above (Fig. 4a). Further, in brainstem nuclei outside the *S. nigra*, for example in neurons of cranial nerve III (CNIII) and the periaquaductal grey, as well as in sections of control cortices anti-parkin clones A15165D, -G and -E also stained vesicular structures adjacent to the nucleus, albeit at a much lesser intensity than pigmented neurons (Tokarew et al., manuscript in preparation).

Intriguingly, sections from younger control subjects (ages, ≤ 33 years) that were processed in parallel revealed less intense, anti-parkin reactivity in *S. nigra* neurons, which matched the paucity of their intracellular pigment (Fig. 8e); of note, mature neuromelanin consistently generates a brown color in sections developed without any primary Ab. The different immunoreactivities seen between younger *vs.* older midbrains suggested that the three anti-parkin clones (A15165D, -G and -E) likely reacted with an age-related, modified form of parkin in situ*,* because the *PRKN* gene is already expressed in dopamine cells at a young age (Fig. 1b; Supplementary Fig. 1a–d, online resource).

To confirm the specificity of the new anti-parkin clones, we serially stained midbrain sections from a 71 year-old, male ARPD patient, who was entirely deficient in parkin protein due to compound heterozygous deletions of *PRKN* exons 2 and 3 (Fig. 8f; Supplementary Fig. 9a–c, online resource) [38]. Development of serial sections with anti-parkin clones A15165E, -D and -G revealed no immunoreactivity in surviving midbrain neurons of the *S. nigra* from this ARPD subject. In the absence of parkin, there was no signal overlap between eDAB reactivity (black color) and either intracellular neuromelanin granules in surviving dopamine cells or with extracellular pigment (brown; Fig. 8f; Supplementary Fig. 9c, online resource). In parallel, development of midbrain sections from individuals with the diagnoses of dementia with Lewy bodies*,* of non-*PRKN-*linked, sporadic PD as well as of cases with incidental Lewy bodies readily demonstrated eDAB reactivity overlapping with neuromelanin for all three anti-parkin clones (Supplementary Fig. 9d–g, online resource; and data not shown). These results demonstrated that the staining by the three anti-parkin clones in our microscopy studies of *post mortem* human brain appeared specific.

### Parkin frequently localizes to LAMP-3^+^-lysosomes within *Substantia nigra* neurons

Neuromelanin granules have been shown to occur in specialized autolysosomes [111]. When screening for co-localization of parkin reactivity with a variety of markers for subcellular organelles in sections of adult control brain, we detected that immunofluorescent signals by anti-parkin (green) and anti-CD63/LAMP-3 (red) antibodies strongly overlapped with pigmented granules of nigral neurons (Fig. 8g–i; see also Supplementary Fig. 9h, online resource).

Using confocal microscopy, we demonstrated that in adult midbrain anti-parkin signals, as generated by clone A15165E, and neuromelanin granules were frequently surrounded by circular, ~ 2 μM (in diameter)-sized rings of anti-LAMP-3 reactivity (Fig. 8i, j). A z-stack video for the parkin and LAMP-3 co-labelling studies is appended (Supplemental Information_video, online resource). We concluded that in the adult, human midbrain from neurologically healthy controls and in surviving neurons of subjects, who suffer from parkinsonism that is not linked to bi-allelic *PRKN* deletion, a pool of parkin appears physically associated with neuromelanin pigment in close association with juxtanuclear, lysosomal structures.

## Discussion

Here, we demonstrate that posttranslational modifications of parkin contribute to its age-related decline in solubility, and in exchange, to redox homeostasis in the human brain. Our study also provides insights into the native processing of the PD-linked parkin protein in the adult midbrain. Parkin’s progressive insolubility in the ageing human brain is relatively unique when compared to other PD-linked proteins and several other cellular constituents, which include mitochondrial proteins. It is also tissue and species-specific. Unlike in the brain, approximately 50% of detectable parkin remain soluble in the spinal cord and in skeletal muscle from aged human subjects, and a comparable loss of parkin solubility is not observed in aged rodent brain and adult monkey cortex (Fig. 1a–d, i; Supplementary Fig. 1d, online resource).

In human control brain, the loss of parkin solubility in *post mortem* tissue correlates with a rise in H_2_O_2_ concentrations and with age, but not with the subject’s sex or the length of PMI (Figs. 1e, j, 2a–c). Although we have analyzed autopsy material with a PMI as short as 2 h (Supplementary Table 1, online resource), in future work we will also extend our efforts to the analysis of specimens removed from living subjects during neurosurgical procedures. Using our cohort of specimens, we found that the transition to parkin insolubility in frontal lobe cortices occurs between the ages of 28 and 42 years (Fig. 1b; Supplementary Fig. 1a–b; Supplementary Table 1, online resource). The age at which parkin transitions in the *S. nigra* will require a larger number of midbrain specimens from young, neurologically normal subjects. While we were unable to assess its solubility in midbrains from subjects younger than 20 years, parkin’s relative distribution in adult midbrain specimens matched the results of control cortices (Fig. 1b). Of note, in the brainstem nuclei that we examined (*i.e., S. nigra; L. coeruleus;* red nucleus; CN III nucleus; periaqueductal grey), we found that parkin’s distribution was not visibly affected by disease state per se (11 control cases *vs.* 9 neuropathological cases; Fig. 1b; Supplementary Table 1, online resource). However, parkin’s total abundance was lower in the *S. nigra* of cases from subjects with various forms of neurodegenerative illnesses, as expected (not shown). In mice, brain parkin showed partial partitioning when oxidative stress had been induced systemically, either acutely or chronically (Fig. 2c–i). In future work, we will examine parkin distribution in larger numbers of brainstem specimens of autopsy material with different neuropathological diagnoses.

In accordance, we demonstrate that a key contributor to parkin insolubility is thiol-oxidation and that the resulting, posttranslational modifications are linked to three protective outcomes: (i) the neutralization of a range of potentially toxic, pro-oxidant radicals (ROS, RES); (ii) the effective lowering of H_2_O_2_ concentrations, including its direct reduction in vitro*;* and (iii) the apparent effect that parkin has on dopamine metabolism through Cys95-mediated conjugation of its radicals and enhanced melanin formation. We have modeled parkin’s redox chemistry-based function in vitro, in cells and in mice, and provide evidence that these outcomes are physiologically relevant to the human brain. From these observations, we propose that insoluble parkin represents a functionally important protein of the ageing human brain including the *S. nigra*. Further, our findings integrate the early literature related to parkin mutations and stress-induced modifications vis a vis its insolubility, which included a wide range of complementary investigations [6, 9, 28, 29, 60, 92, 98, 99, 103, 104], such as findings from induced pluripotent stem cell-derived, human dopamine neurons [32, 35, 68]. Our discovery of a function for parkin in redox homeostasis also helps explain seemingly disparate evidence of previous observations made in studies of flies, mice [70, 74] and humans [73].

The reactivity of cysteine thiols is governed by their own redox state as well as by the surrounding electrostatic environment, which includes the charges of neighbouring residues [105]. Unlike parkin, 34 out of 35 cysteines found in BSA are engaged in disulphide bonds [37, 72]. BSA was not able to reduce H_2_O_2_, nor did it enhance the formation of insoluble melanin polymers in vitro under these conditions. Two other Zn^2+^-coordinating, cysteine-containing proteins that we tested, RNF43 and HOIP^CD^ (Fig. 5c), also did not lower H_2_O_2_, thus suggesting that select cysteines in parkin have a high affinity for ROS and, as discussed below, RES molecules. When mapping the redox state of parkin cysteines under progressively pro-oxidant conditions in vitro, we found that Zn^2+^-coordinating residues at its RING domains are not protected from modifications by ROS [56] (Supplementary Table 2, online resource). This observation suggests that oxidative changes of parkin in vivo could occur continuously in the form of a gradient*,* rather than representing a binary event.

Based on our results, we also estimated the levels of pro- vs*.* anti-oxidant forces. There, the ratio of H_2_O_2_-to-r-parkin (0.1–1.0 mM of H_2_O_2_ per 1 ng of r-parkin) was within the physiological range of what we had measured for human control brain extracts (i.e*.,* 0.4–6.0 mM of H_2_O_2_ per 1 ng of parkin). In the latter, H_2_O_2_ concentrations were calculated to lie between 0.7 and 9.1 mM/mg of tissue (see Supplementary Table 1, online resource). Using semiquantitative Western blotting with aliquots of the same Ab lot (Prk8), parkin concentrations were estimated to be ~ 1.42 ng/mg brain tissue using r-parkin dilutions as standards; these had been run in parallel with brain lysates of ARPD cortices to demonstrate specificity for the detection of the ~ 51–53 kDa holoprotein. These estimates represent a first approximation of the concentration of wild-type parkin in the adult human brain; these numbers may need to be revised in the future based on controlling for potentially confounding variables, such as the presence of truncated species and modified forms (not detected by our antibodies), and/or due to marked variability in parkin’s turnover rate in different regions of the cortex and between subjects.

As was observed for r-parkin, we also found cysteine residues that were oxidized in parkin proteins after their affinity isolation from human control cortices and mouse brains, including of Zn^2+^-binding ones. For example, Cys253 (Cys252 in mice), which helps coordinate Zn^2+^ within parkin’s RING1 domain, was frequently identified by us as being oxidized (Figs. 3i, 4e, g). We predict that variable modifications of non-Zn^2+^-coordinating residues in human parkin could induce early, conformational changes in parkin’s tertiary structure, such as at Cys95, which is located in the—heretofore structurally understudied—linker region, or Cys59, as positioned in its ubiquitin-like (UbL) domain [17] (see Figs. 6e–h, 7c–g). Such N-terminally located changes could profoundly affect both the structure and function of other domains in wild-type parkin, as has been convincingly delineated in studies of parkin’s E3 ligase activity as a readout following modifications at its UbL domain [7, 8, 51, 60, 69, 71, 101, 104, 107] (and reviewed by Yi et al. [108]*.*). Our results do not exclude the possibility that other non-thiol-based, posttranslational modifications alter parkin’s solubility, such as phosphorylation at Ser65 [46], or at Ser77 [17], which we found in brains of MPTP-treated mice. Currently, ongoing experiments seek to answer the question as to how structural changes caused by select ARPD-linked parkin mutants, e.g*.,* p.C431F and p.G328E, as determined by far-UV-circular dichroism, dynamic-light scattering and NMR techniques, could alter redox functions in vitro. Their completion will add to our understanding as to how these mutants alter solubility and half-life of nascent parkin proteins in cell-based studies [9, 92].

As mentioned above, *PRKN*-linked ARPD is thought to be pathologically restricted to catecholamine producing cells of the brainstem [15, 40, 45, 53, 55]. Dopamine neurons of the *S. nigra* have unique biophysical properties that lead to high bioenergetic demands and the related rise in oxidative stress [23]. Further, unlike in other animals, dopamine is not completely catabolized in the human brain, and neuromelanin is thought to be essential for the sequestration and long-term storage of its otherwise toxic metabolites [110]. We found parkin to be involved in mitigating two well-established, PD-linked stressors (i.e*.,* ROS; dopamine radicals), which is indirectly supported by our findings in the human brain.

We show that parkin functions as a classical redox molecule that is able to lower H_2_O_2_ in a thiol-dependent manner. In the absence of wild-type parkin, H_2_O_2_ concentrations are elevated in the human brain (Fig. 5i), in dopaminergic cells (Fig. 5j, k) and in brains from mice exposed to MPTP-toxin (Fig. 5h). There, acute MPTP exposure not only correlated with a decline in parkin solubility but also with the oxidation of select cysteines (Fig. 4a). Hence, *PRKN* expression contributes to *anti-oxidant activity *in vivo through a net reduction in H_2_O_2_ levels*,* which can occur in part through its direct reduction, as shown by us in vitro (Fig. 5; Supplementary Fig. 5, online resource).

Because both MPTP toxin exposure and *Sod2* gene function affect mitochondrial integrity [12, 20], we reason that redox homeostasis in the cytosol, as coregulated by parkin oxidation, could also indirectly influence the health of mitochondria, in addition to E3 ligase-associated mitophagy (and MITAP). Such a cross-talk between cytosol and mitochondria likely includes glutathione metabolism-linked pathways, in which we and others found parkin cysteines to be involved in as well [11, 17, 25, 34, 79, 89].

A role for *PRKN* expression in the neutralization and sequestration of dopamine metabolites may explain why dopamine synthesizing neurons are at greater risk in humans born with parkin deficiency. Previously, parkin has been shown to be uniquely sensitive to dopamine stress leading to aggregate formation [51, 104] (Supplementary Fig. 6a, b, online resource). In both cells and mice, *prkn* gene expression has been indirectly implicated in the metabolism of this neurotransmitter, in particular under ex vivo conditions, such as induced by high dopamine level-induced stress [25, 34–36, 43, 51] (see also Supplementary Fig. 6c, d, online resource).

Our results, and those by others, suggest that dopamine-mediated stress in neural cells is ameliorated when parkin undergoes irreversible modifications by dopamine metabolites. However, in contrast to current interpretations, which stipulate oxidation by quinones is equal to a loss of parkin activity, we posit that such oxidation is part of parkin’s physiological role within post-mitotic cells of the adult brain based on two principal findings. First, we demonstrate that wild-type parkin directly interacts with highly electrophilic dopamine metabolites at specific residues, foremost Cys95 (Fig. 6e–h). This primate-specific cysteine is located within the linker region next to charged residues that impact its electrostatic properties and likely its redox reactivity [24, 105]. In support, we found that in addition to dopamine adduct conjugation, Cys95 is vulnerable to ROS attacks (Figs. 3h,  4b, c), and in parallel studies, is S-glutathionylated when exposed to rising concentrations of oxidized glutathione [17]. Strikingly, we found that Cys95 is not only required for parkin-dependent enhanced melanin formation, but also for participation in effective H_2_O_2_ reduction in M17 cells during dopamine stress-mediated toxicity (Figs. 6e–g,  7f–g).

Second, our finding that parkin augments melanin formation in vitro, together with our finding that the protein is closely associated with neuromelanin granules within LAMP-3^+^- lysosomes of human brain (Fig. 8g–j; Supplementary Fig. 9h, online resource), suggest a role for parkin in *dopamine metabolism-linked neuroprotection* (Supplementary Fig. 10, online resource). We have noted with interest that several autopsy reports have described lesser neuromelanin content in surviving neurons of the *S. nigra* in *PRKN*-linked ARPD [22, 27, 30, 95, 106, 109] (Fig. 8f). Intriguingly, variants at the *LAMP3/CD63* locus, as well as of other dopamine metabolism-related genes, e.g*.*, *GCH-1,* have been recently identified as modifiers of susceptibility to late-onset, typical PD [33, 66, 100]. However, proof of the concept that parkin plays an important, contributing role in the formation of neuromelanin in human brain awaits a suitable animal model.

To date, parkin is best known for its function as an E3 ligase and the ubiquitin ligation-dependent involvement in mitophagy. Because ubiquitin-ligating activity occurs via cysteine-mediated trans-thiolation, controlling the cellular redox state and functioning as an E3 ligase may not be mutually exclusive. For example, low concentrations of pro-oxidants, as well as sulfhydration, can activate parkin’s E3 activity in vitro [71, 97, 107]. A similar duality in functions, i.e., regulating ubiquitylation and redox state in cells, has been previously described for the sensitive-to-apoptosis gene (SAG) product, also known as RBX2/ROC2/RNF7 [93, 94]. It contains a RING finger, and similar to parkin, was found to form HMW oligomers through oxidation of its cysteines [93, 94]. SAG protein protects cells from oxidative stress in a thiol-mediated manner in addition to functioning as an E3 ligase.

From this analogy, we postulate that parkin’s *cytoprotective E3 function* and its role in mitophagy are possibly linked to its soluble form within the cytosol, which could be most important during early developmental stages, such as during organ development [26], in dividing striated muscle cells [80], and in relatively younger, neural cells including glia [89]. In support, Yi et al. recently described a strong correlation between parkin point mutants, their impact on structure and protein stability *vs.* ubiquitin ligase activity and the degree of mitophagy efficiency [108]. Conversely, redox-based neutralization of radicals by wild-type parkin could be more essential to the sustained health of long-lived, postmitotic cells, e.g.,* S. nigra* neurons.

In summary, we have shown that parkin fulfils criteria of a typical redox molecule: the sensing of oxidative (and reducing) stress via its thiols; and the direct, reciprocal redox regulation of its environment, thus conferring protective outcomes. If confirmed by future work, this redox chemistry-based expansion of parkin functions in the ageing human midbrain (Supplementary Fig. 10, online resource) may open the door to testing its anti-oxidant role in related neurodegenerative conditions, such as late-onset, non-*PRKN*-linked PD [13]. Most important, our findings emphasize the need for early identification of persons afflicted by bi-allelic *PRKN* gene mutations for the prioritization of appropriate interventions in the future, such as via gene therapy [44] and polyvalent, anti-oxidant therapy [78].

## Supplementary Information

Below is the link to the electronic supplementary material.

Supplementary file1 (PDF 2107 KB)

Supplementary file2 (PPTX 7815 KB)

## Data Availability

Original data associated with this study are available in the main text and supplementary figures and tables; additional data will be made available upon request.
