# Supplementary material for: Age-associated insolubility of parkin in human midbrain is linked to redox balance and sequestration of reactive dopamine metabolites
Source: Acta Neuropathol. 2021 Mar 10;141(5):725–54. doi: 10.1007/s00401-021-02285-4 (PMC8043881; doi:10.1007/s00401-021-02285-4)
Supplement: Supplementary file 1 — Supplementary file1 (PDF 2107 KB) [file 401_2021_2285_MOESM1_ESM.pdf]

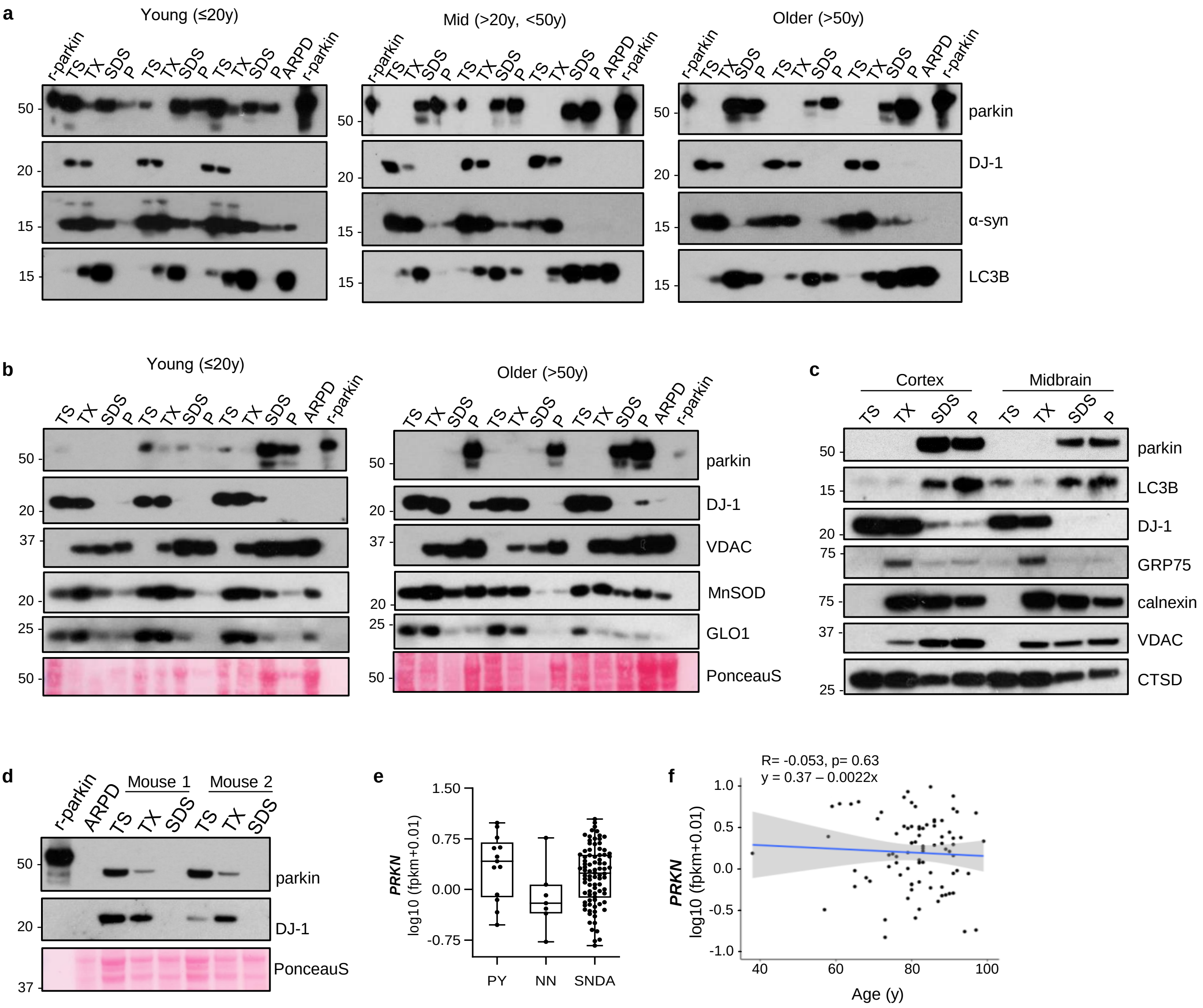

Supplementary Figure 1: Parkin becomes largely insoluble with progression in age in *post mortem* human brain.
(**a**) Western blots of parkin, DJ-1, α-synuclein and LC3B distribution in 9 representative human cortices (see **Supplementary Table 1**). Tissue fractionation and age ranges were as described in **Fig. 1**; SDS/PAGE experiments run under reducing conditions; SDS-extracted fractions of parkin-deficient PD cortices (ARPD) and r-parkin are included as controls.
(b) Western blots of parkin, DJ-1, VDAC, MnSOD and glyoxalase-1 proteins, and Ponceau S staining in serially fractionated human cortices from younger (n=3) and older (n=3) individuals. Quantification of relative protein distribution is shown in **Fig. 1g**.
(c) Western blot of indicated proteins from serially fractionated cortex and midbrain from a single donor as described in (a).
(d) Quantification of log-transformed *PRKN* mRNA signals from individual pyramidal neurons (PY), leukocytes (non-neuronal cells; NN) and *S. nigra* dopamine neurons (SNDA) isolated from *post mortem* control brains (age range, 38 to 99 yrs).
(e) Linear regression analysis of log-transformed *PRKN* transcripts as a function of age in human control *S. nigra* dopamine neurons where each dot represents values for a single neuron, as shown in (**d**).

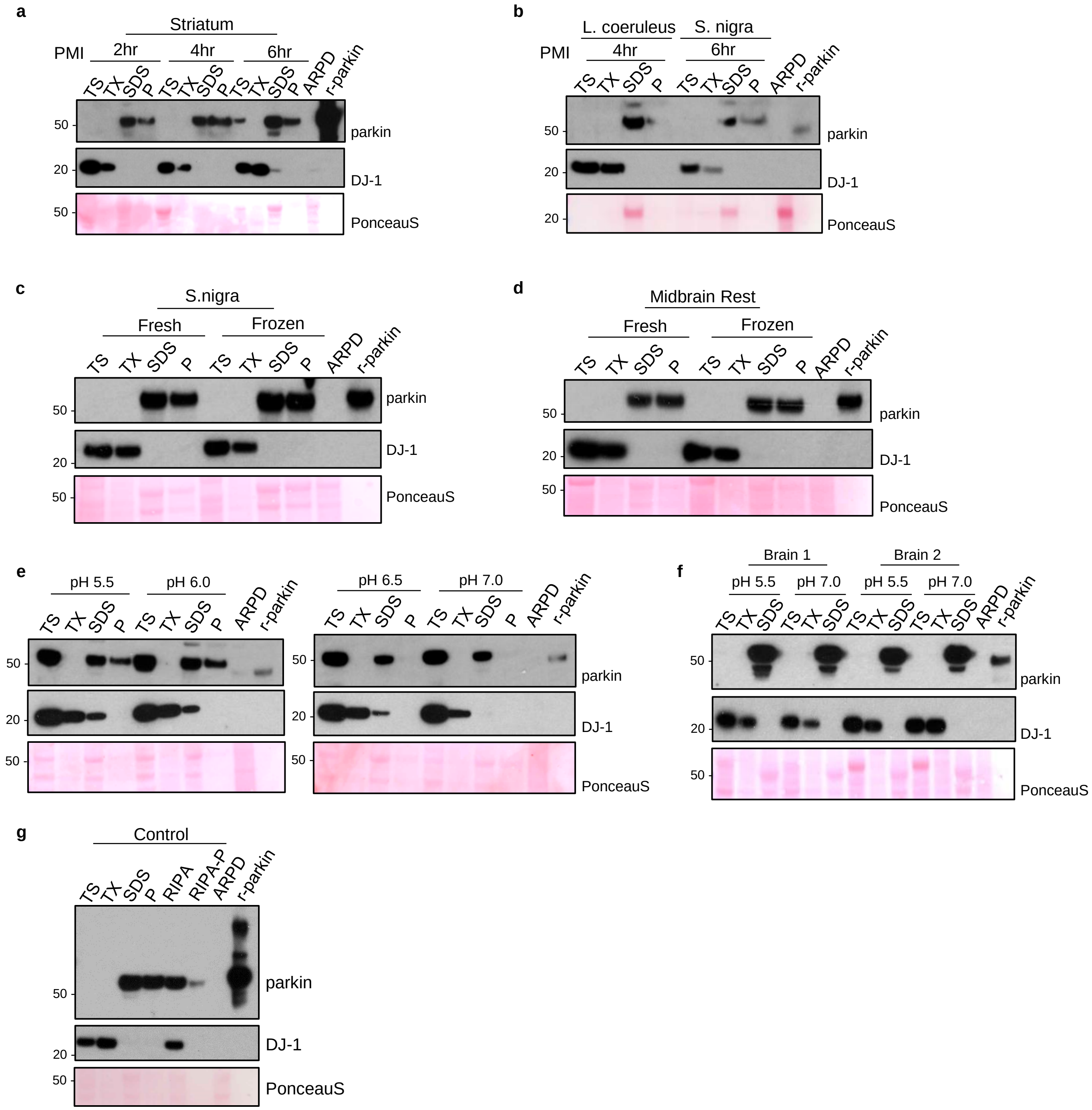

Supplementary Figure 2: Parkin solubility in human brain is not detectably altered by length of *post mortem* interval, tissue freezing, or pH levels of the buffer.

(a-b) Western blots of parkin and DJ-1 distribution as well as Ponceau S staining for fractions of human brain tissue from striatum **(a)**, *L. coeruleus* and *S. nigra* **(b)** with short *post mortem* interval (2-6 hrs, as indicated).

(c-d) Western blots, as described in (a), from dissected *S. nigra* **(c)** and posterior midbrain structures comprising nucleus of cranial nerve-III and the periaqueductal grey (**d;** rest). Tissues were collected *post mortem* and parkin distribution visualized in aliquots of the same specimens processed in parallel after being kept at 4°C or processed via one-time freezing to -80°C and subsequent thawing prior to serial fractionation.

(e-f) Western blots of parkin and DJ-1 distribution as well as Ponceau S staining in fractions of human cortex (**e**, single brain; **f**, two different brains) serially extracted in parallel using standard buffers with varying pH, as indicated.

(g) Western blots of parkin and DJ-1 distribution in a human cortex sample following serial fractionation with TS- TX-, SDS- and Pellet buffers compared to processing by standard RIPA buffer, where the pellet after RIPA extraction is denoted as RIPA-P.

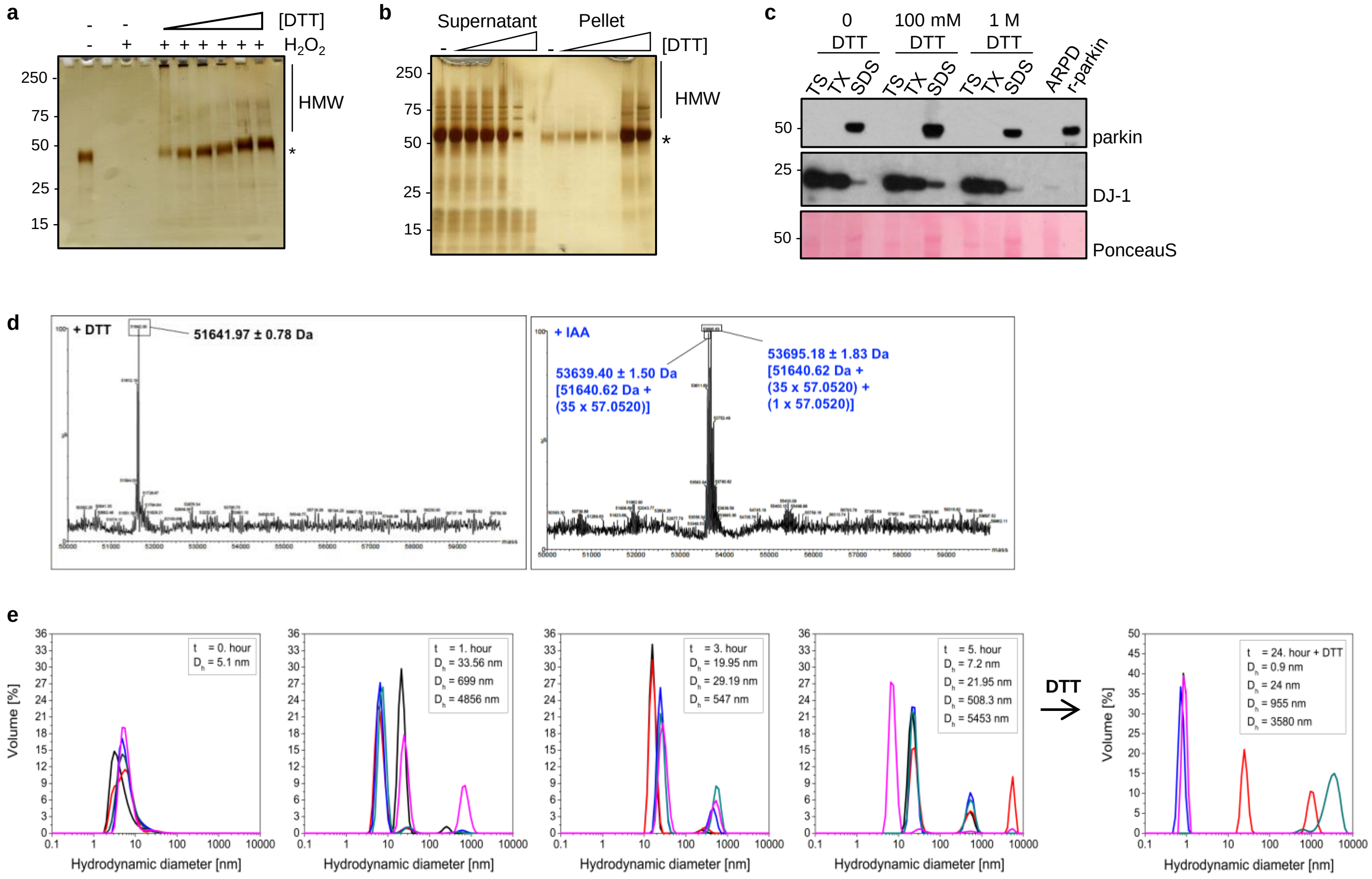

Supplementary Figure 3: Oxidation of human parkin thiols promotes insolubility.

(a) Silver stained gel of wild-type, human r-parkin exposed to H_2O_2 (10 mM), followed by treatment with increasing concentrations of DTT (0-100 mM) prior to centrifugation and loading of the supernatant onto SDS/PAGE, run under non-reducing conditions.

(b) Detection of r-parkin in soluble (supernatant) and insoluble phases (pellet; recovered by 10% SDS-containing buffer) following exposure to increasing concentrations of DTT (0-1M), run under non-reducing conditions.

(c) Western blot analyses with anti-parkin and anti-DJ-1 as well as Ponceau S staining of three dissected pieces from a single human cortex specimen serially extracted in parallel by TS-, TX- and SDS-buffer without (O DTT) or including either 100 mM or 1M DTT in the extraction buffers, as indicated, run under reducing conditions.

(d) Spectra from LC-MS/MS analyses of recombinant (r-), human, wild-type parkin holoprotein (without any trypsin digestion) without pre-labelling (panel on the left) and after tagging of 35 *vs.* 36 thiol-carrying residues by iodoacetamide (IAA; right panel), corresponding to the three main peaks (one in left panel; two in right panel), as indicated. The 51,641.97 Da peak closely matches its calculated mass of 51,640.62 Da; 53,639.40 Da corresponds to the conjugation of 35 IAA adducts; 53,695.18 Da corresponds to 36 IAA adducts, indicating that all 35 cysteine residues and either the N-terminal amino group or a single methionine residue was IAA-modified.

(e) Dynamic light scattering analysis showing progressive size changes, as measured in hydrodynamic diameters (nm), as monitored during 0, 1, 3 and 5 hrs at room temperature. The structural state for wild-type, human r-parkin under non-reducing, native conditions showed increased aggregate formation over time, which was partially reversed by DTT.

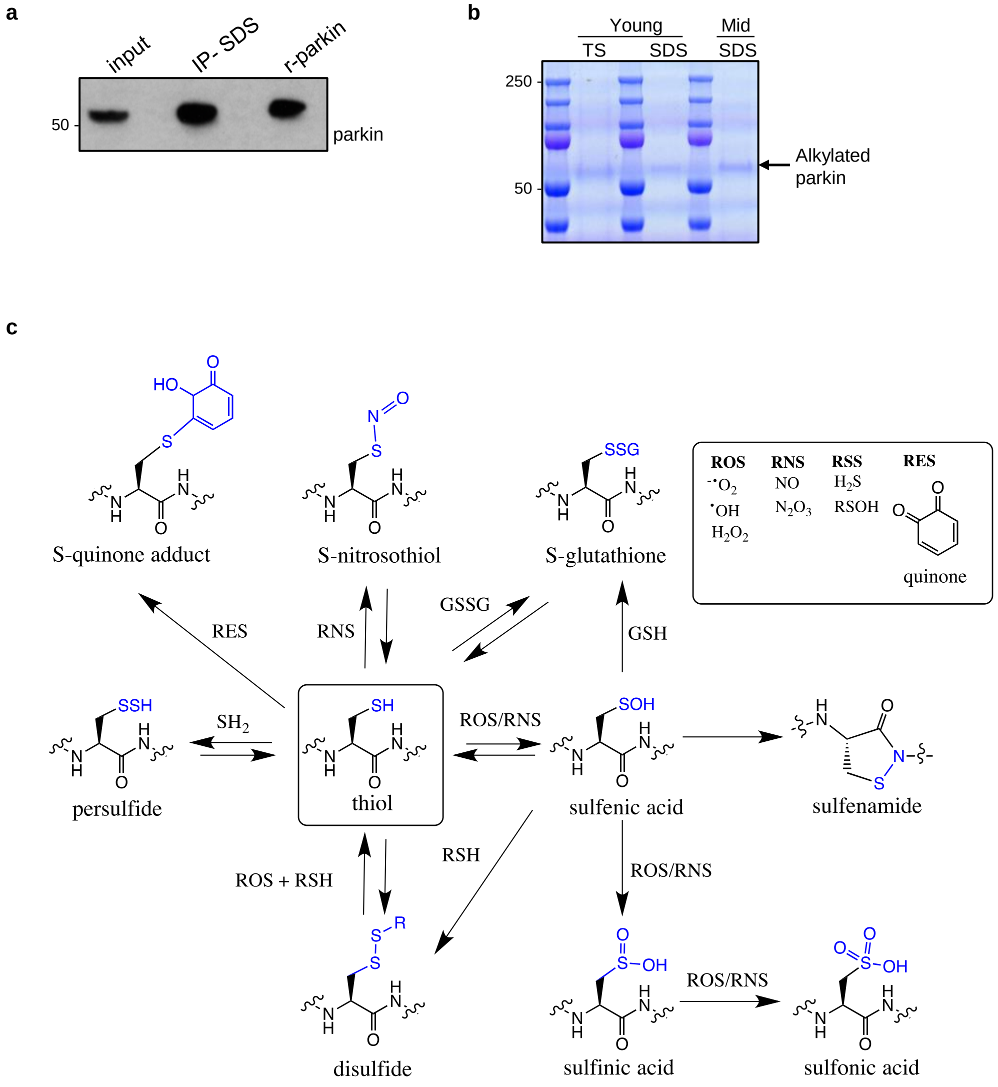

Supplementary Figure 4: Immunoprecipitation of parkin from human brain and summary of redox-related thiol chemistry.

(a-b) Representative Western blot **(a)** and Coomassie blue-stained **(b)** visualization of parkin immunoprecipitated from human frontal lobe cortex, as described [Shimura and Schlossmacher, Methods Enzymol 2005] by monoclonal anti-parkin A15165-B and visualized by polyclonal anti-parkin 2132, in preparation for LC-MS/MS (see also **Fig. 4**). Brain tissue was homogenized in the presence of IAA to prevent the oxidation of reduced thiols during processing, thereby generating alkylated-parkin monomers at the 51-54 kDa position.

(c) Schema of select, reversible and irreversible cysteine modifications that can occur on thiols (-SH) due to attacks by reactive oxygen species (ROS), reactive nitrogen species (RNS), reactive sulfur species (RSS) and reactive electrophilic species (RES), which include dopamine quinones. Graphic summary was modified from Alcock *et al.*, 2018.

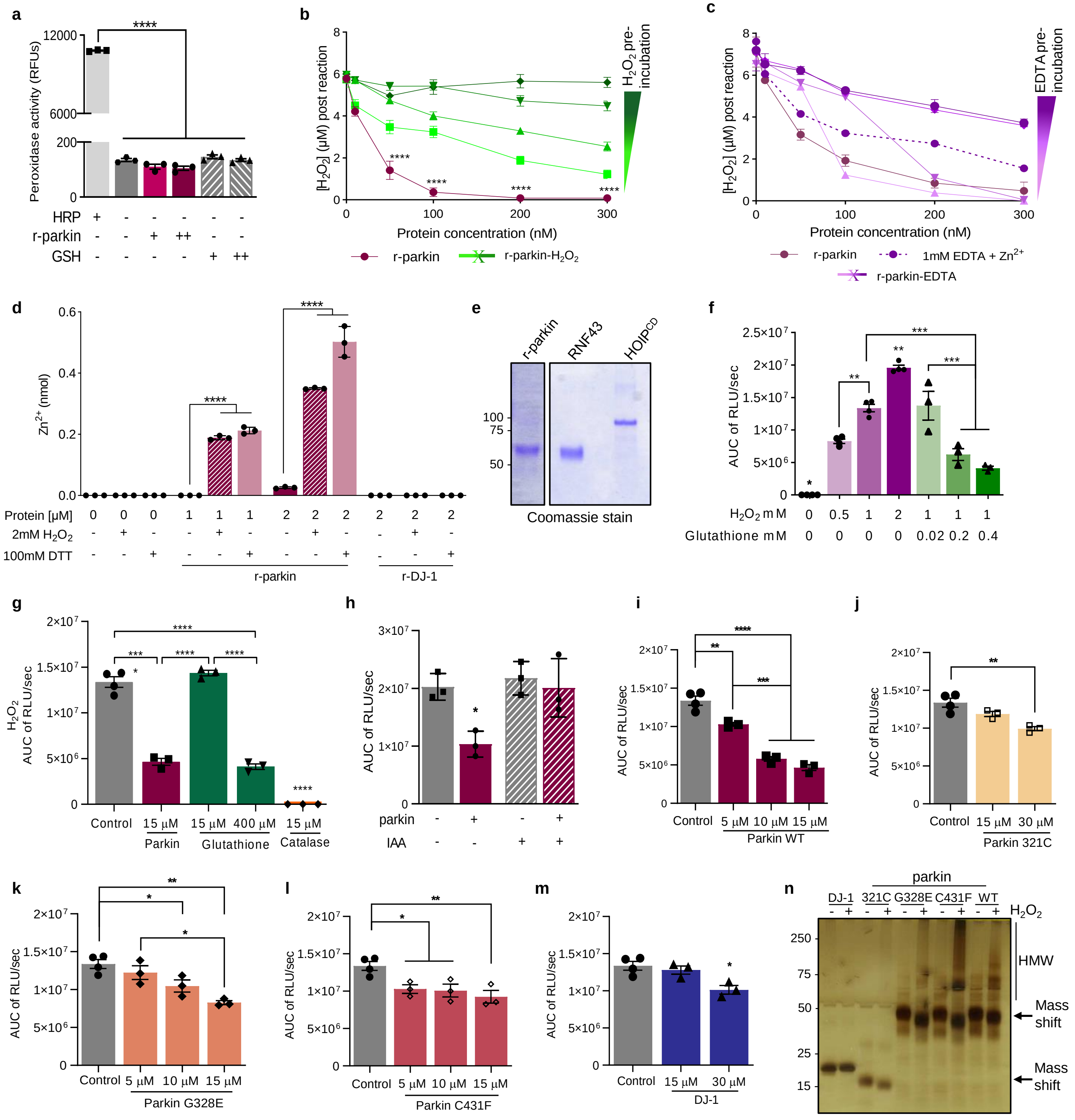

Supplementary Figure 5: Parkin directly reduces hydrogen peroxide in a concentration- and thiol integrity-dependent but non-enzymatic manner.

Please see legend on next page.

Supplementary Figure 5: Parkin directly reduces hydrogen peroxide in a concentration- and thiol integrity-dependent but non-enzymatic manner.

(a) Peroxidase enzymatic activity for r-parkin and glutathione (GSH; +, 0.5µM; ++, 1µM), as tested *in vitro* in comparison to horseradish peroxidase (HRP, 1mU/mL). Mean peroxidase activity ± SEM. ****p<0.0001 by 1-way ANOVA with Tukey's post hoc test; [F (5, 12) = 81945, p< 0.0001].

(b-c) Quantification of H_2O_2 concentrations by AmplexRed following incubation of increasing levels of r-parkin **(b)** pre-oxidized with increasing concentrations of H_2O_2, or **(c)** treated with increasing concentrations of EDTA or EDTA with excess zinc ions, as indicated. A two-way ANOVA with Tukey's post hoc test (****p<0.0001) was used for statistical analysis; b, [F (20, 60) = 24.37, p< 0.0001]; and c, [F (25, 12) = 6.438, p= 0.0008].

(d) Quantification of unbound zinc (Zn^{2+}) ions quantified using an *in vitro* assay. Increasing concentrations of recombinant, wild-type parkin and DJ-1 (as control) proteins were assayed under basal conditions or under oxidizing (2 mM H_2O_2) *vs.* reducing (100 mM DTT) conditions. Free Zn^{2+} release was measured colorimetrically at OD560 nm. A two-way ANOVA with Tukey's post hoc test (****p<0.0001) was used for statistical analysis; b, [F (4, 18) = 151.1, p< 0.0001].

(e) Coomassie Blue-stained visualization of r-parkin, RNF43 and $HOIP^{cd}$ proteins, used in the AmplexRed assay shown in **Fig. 5c**.

(f) Area under the curve (AUC) plots from kinetic readings of an *in vitro* colorimetric H_2O_2 assay comparing increasing concentrations of ROS input (shades of green) and the effect of rising concentrations of GSH (shades of purple). AUC integrated the total value of H_2O_2 signals generated over a 10 min-long time course in the assay. Results analyzed by one-way ANOVA [F (6, 18) = 73.02, p< 0.0001]

(g-m) AUC graphs for results from *in vitro* H_2O_2 assays for various concentrations of recombinant proteins, as indicated. Statistical analysis was performed as in **Fig. 5e**. Results analyzed by one-way ANOVA, (**f**) [F (4, 11) = 229.6, p< 0.0001], (**g**) [F (3, 8) = 7.415, p= 0.0107], (**h**) [F (3, 9) = 87.58, p< 0.0001], (**i**) [F (2, 7) = 13.45, p=0.0040], (**j**) [F (3, 9) = 11.23, p= 0.0021], (**k**) [F (3, 9) = 7.263, p= 0.0089] and (**l**) [F (2, 7) = 8.536, p= 0.0133]

(n) Visualization of recombinant PD proteins post H_2O_2 exposure by silver staining where SDS/PAGE gel was run under non-reducing conditions.

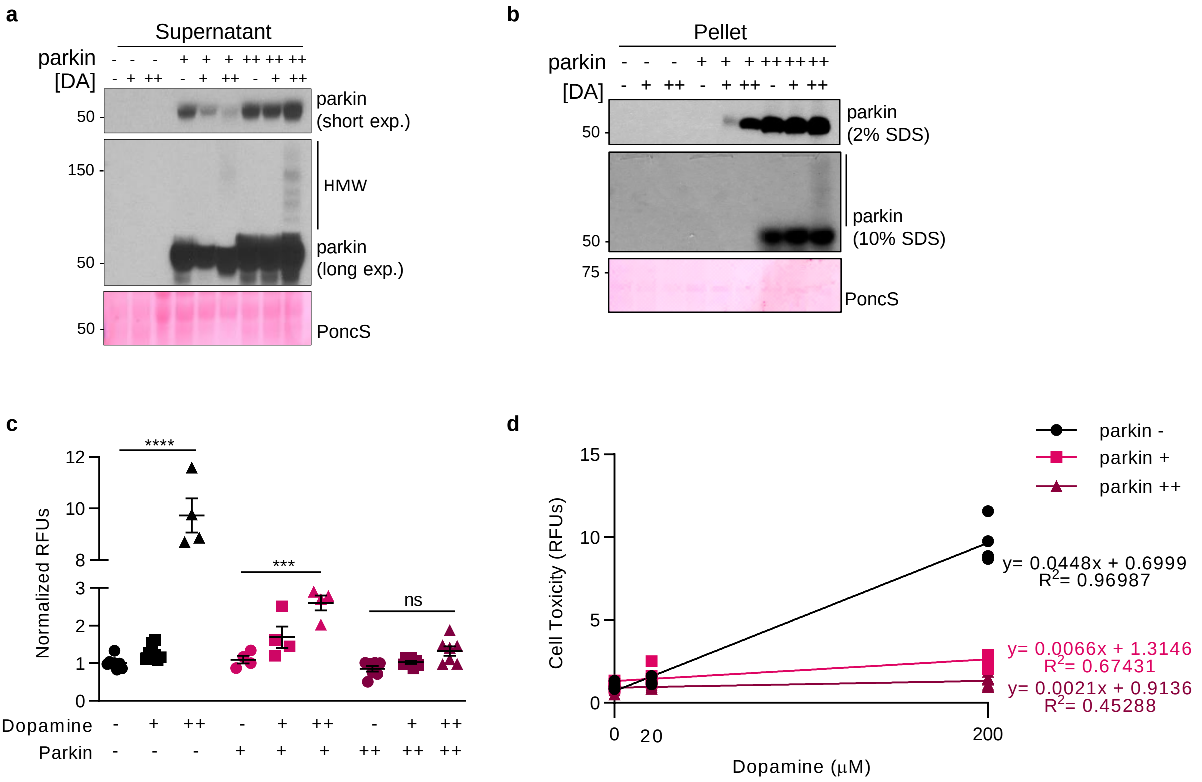

Supplementary Figure 6: Wild-type, human parkin protects neural cells from dopamine toxicity in a protein concentration-dependent manner.

(a-b) Western blots of parkin in the soluble supernatant (**a**) and insoluble, serial pellet (**b**) fractions of lysates from dopamine-treated human M17 neuroblastoma cells, which stably express vector-control plasmid (parkin -) or myc-tagged, human *PRKN* cDNA at mid- (+) or high (++) levels. Cells were exposed to 20 µM (+) and 200 µM (++) dopamine for 20 hrs, as indicated. SDS/PAGE gels were run under reducing conditions.

(c) Cell viability assay of cells highlighted in (a, b). Representative data are shown for the mean of duplicates ± SEM from n=4-8 independent experiments; *p<0.05 by 2-way ANOVA with Tukey's post hoc test; [F (4, 44) = 189.2, p< 0.0001].

(d) Correlation studies of experiments, as conducted in (a, b), to monitor parkin expression levels *vs*. cell survival.

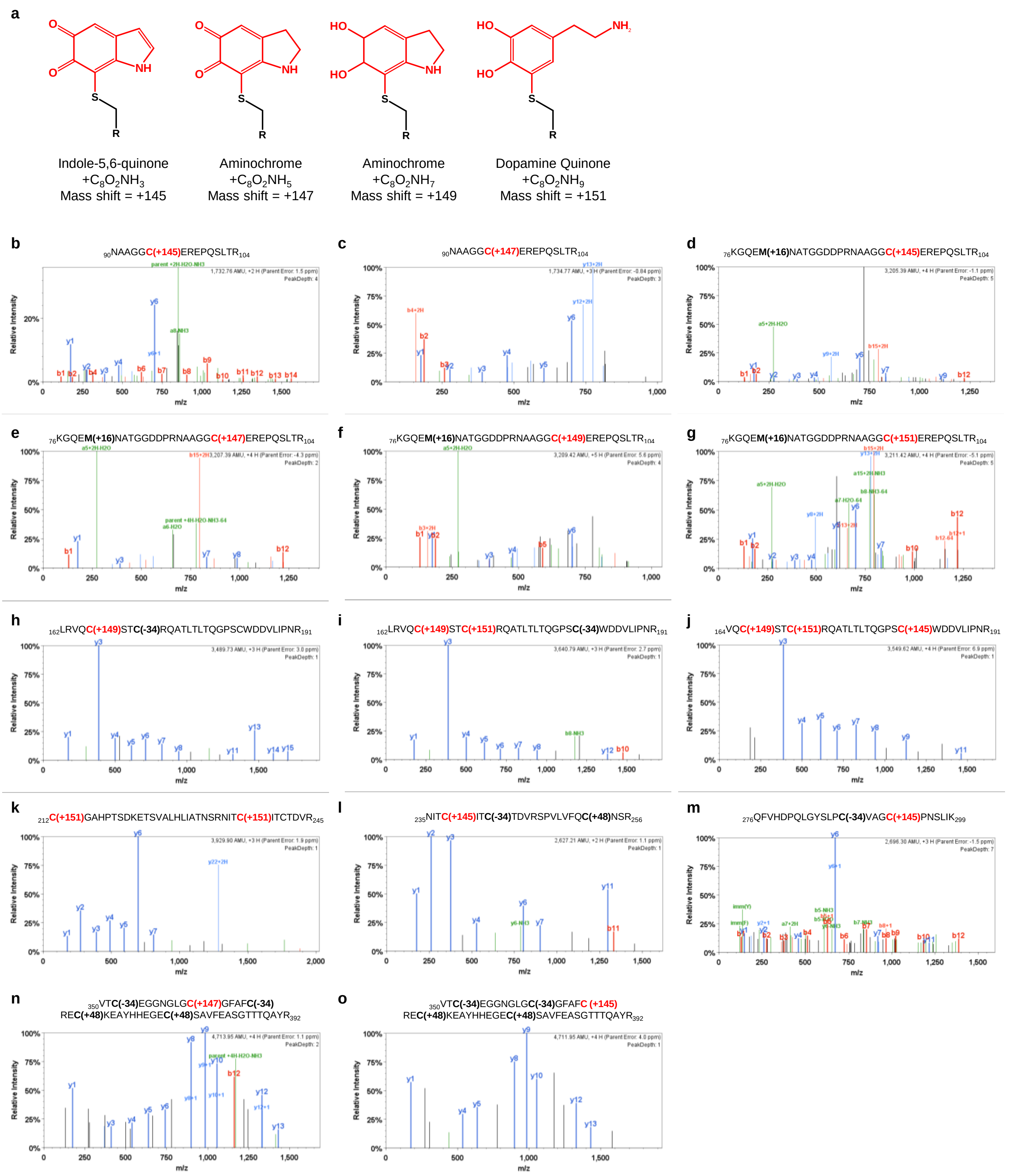

Supplementary Figure 7: Human parkin conjugates dopamine metabolites at cysteine 95 and other cysteine residues.
(a) Chemical structures of 4 dopamine metabolites (red) conjugated to a thiol group (black) that were screened for in LC-MS/MS experiments, with their corresponding mass shift added.
(b-o) LC-MS/MS-generated spectra following trypsin digestion of aminochrome-treated, human r-parkin protein highlighting representative adduct conjugation events, which were identified by mass shift gains as shown in (**a**), at the following residues: Cys95 (**b-g**), Cys166, Cys169 and Cys182 (**h-j**), Cys212 (**k**), Cys238 (**l**), Cys293 (**m**), Cys360 (**n**), and Cys365 (**o**). See also **Fig. 6e,g**.

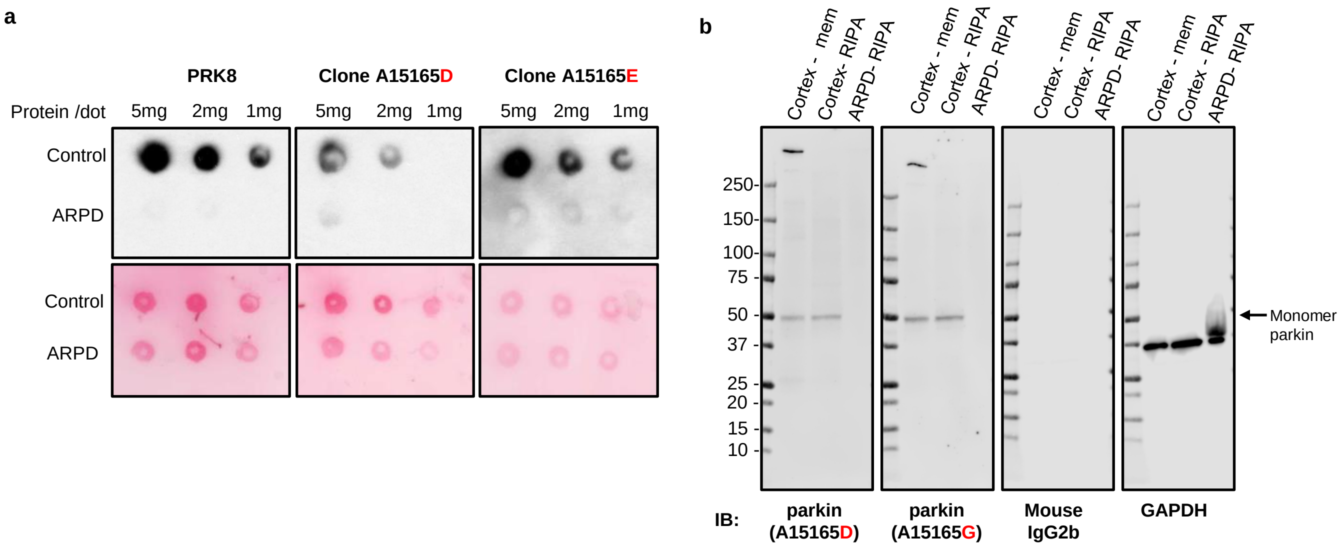

c

Clone	Epitope	Domain
A15165**B**	aa 89-93	Linker
A15165**D**	aa 397-404	REP
A15165**G**	aa 398-404	REP
A15165**E**	aa 177-184; aa 443-453; aa 459-464	RING0; RING2 [conformational]

Supplementary Figure 8: Characterization of four, new monoclonal antibodies raised in mice against human parkin. **(a-b)** Characterization of four murine, monoclonal antibodies (of IgG_2 isotype; clone-B, -E, -D, and -G) by **(a)** non-denaturing dot blots against human brain lysates (SDS fractions from cortices of control individuals and *PRKN*-linked ARPD patients); and **(b)** by denaturing SDS/PAGE under reducing conditions and Western blotting of extracts from cortical specimens of a control brain and a parkin-deficient ARPD case. Screening by these three methods as well as by cell-based microscopy using indirect immunofluorescence (not shown) revealed specific staining for four anti-parkin clones (-B, -E, -D and -G), which was conformation-dependent for clone-E.
(c) Select list of epitopes within the sequence of human parkin, as recognized by clones -B, -E, -D, and -G and identified by screening with overlapping 7-12 amino acid-long peptides covering full-length, human parkin (Tokarew et al., manuscript in preparation).

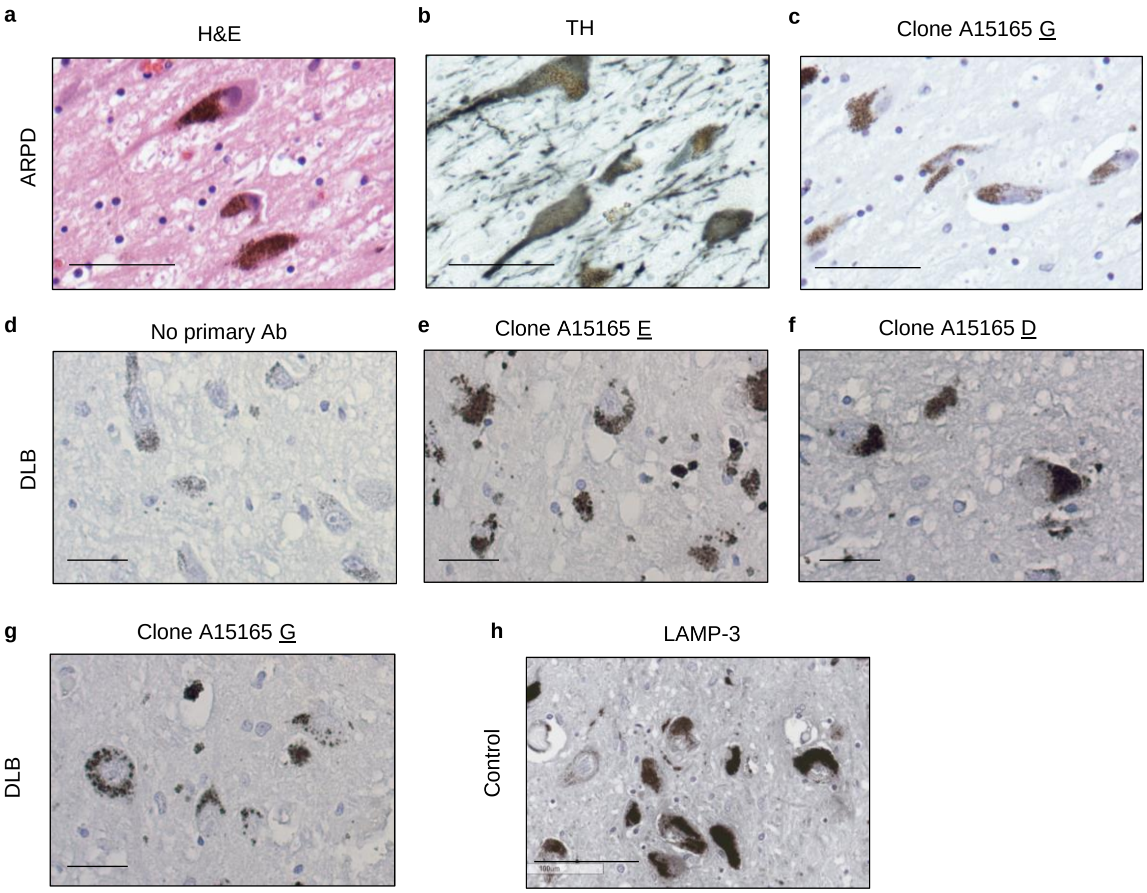

Supplementary Figure 9: Parkin is specifically detected in human midbrain sections by routine immunohistochemistry.

(a-c) H&E **(a)**, anti-tyrosine hydroxylase (TH) **(b)** and anti-parkin (clone A15165-G) (**c**) staining of dopamine neurons in the *S. nigra* of midbrain sections from a parkin-deficient ARPD case [Kano et al., npj Parkinson's Disease 2020].

(**d-g**) Immunohistochemical detection of parkin in the *S. nigra* of an individual with dementia with Lewy bodies. Both intra- and extracellular anti-parkin-reactive neuromelanin granules are visible. **(b)** No primary antibody control and staining with anti-parkin monoclonal antibodies **(e)** A15165-E, **(f)** -D and **(g)** -G are shown.

(h) Immunohistochemical detection of LAMP-3 protein in dopamine neurons of the *S. nigra* from an adult control brain. Scale bars represent 100 µm.

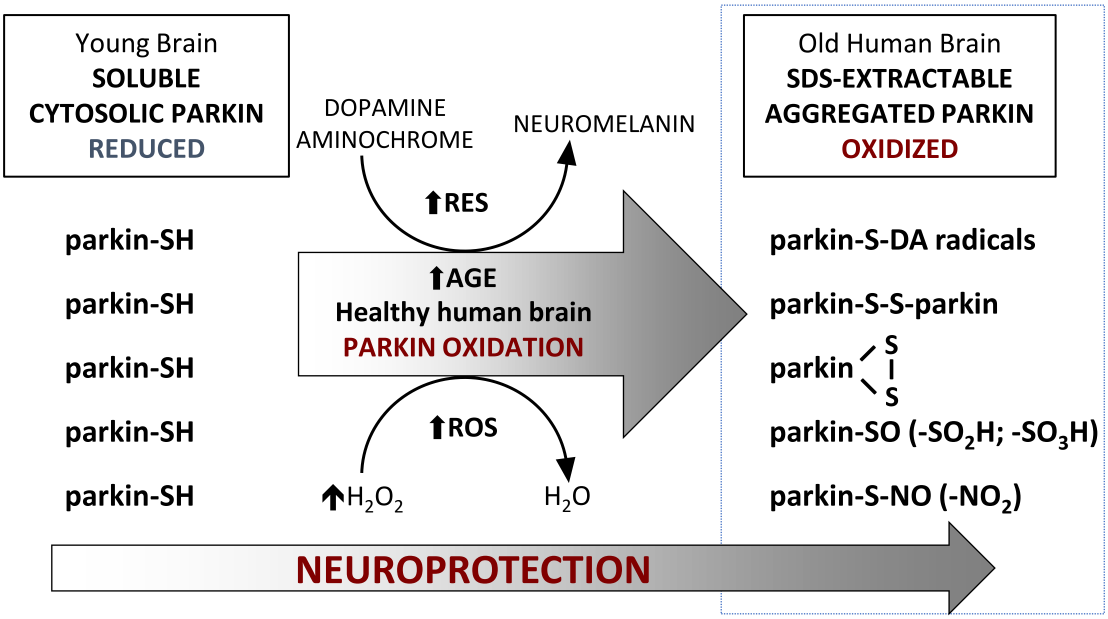

Supplementary Figure 10: Graphic summary of a working model for parkin's redox functions in adult, human dopamine neurons.

In human brain, parkin thiol (-SH) oxidation neutralizes cellular reactive oxygen species (ROS; H_2O_2) and potentially toxic dopamine (DA) radicals (*e.g.*, DA quinones; RES) during normal ageing. In human brain, both reversible and irreversible oxidation events occur gradually, which promote parkin's transition into a less soluble state by the beginning of the 5th decade. In adult dopamine neurons of the *S. nigra*, post-translational modifications lead to the accumulation of a pool of parkin within LAMP-3-positive lysosomes. This multimodal oxidation of parkin confers neuroprotection. In *PRKN*-linked ARPD, the absence of parkin's redox effects contributes to a rise in ROS (and RNS) levels, reduced sequestration of dopamine radicals (RES), and possibly, less neuromelanin formation.

Supplementary Table 1: List of human tissue specimens examined in this study. Please see legend on next page.

Sample ID	Brain region	Age	Sex	PMI (hrs)	Diagnosis	% soluble parkin	H_2O_2/ Tissue ratio	Appears in figures
1	FC	5	F	33	Healthy control	54.1		1b, 1e, 1f, 1g, 1h, 1k, S1a, S1b
2	FC	5	F	20	Healthy control	22.6	2.551	1b, 1e, 1f, 1g, 1h, 1k, 2[a], 2b, 2c, S1a, S1b
3	FC	8	M	5	Healthy control	3.7	1.780	1b, 1e, 1k, 2a, 2b, 2c
4	FC	13	M	13	Healthy control	86.0	2.217	1b, 1e, 1f, 1k, 2a, 2b, 2c
5	FC	15	F	9	Healthy control	27.5	1.349	1b, 1e, 1f, 1k, 2a, 2b, 2c
6	FC	16	F	20	Healthy control	40.6		1b, 1e, 1f, 1g, 1h, S1b
7	FC	16	F	14	Healthy control	51.3	1.016	1b, 1e, 1f, 1k, 2a, 2b, 2c, S1a
8	FC	17	M	23	Healthy control	5.1	0.701	1b, 1e, 1k, 2a, 2b, 2c
9	FC	17	M	22	Healthy control	45.3		1b, 1e, 1k
10	FC	20	F	19	Healthy control	63.3		1b, 1e, 1f, 1k
11	FC	20	M	8	Healthy control	37.5		1b, 1e, 1f, 1k
12	FC	20	M	6	Healthy control	3.6		1b, 1e, 1f, 1k
13	FC	20	M	5	Healthy control	89.4		1b, 1e, 1f, 1k
14	FC	21	M	30	Healthy control	46.1		1b, 1e, 1f, 1k
15	FC	28	M	33	Epilepsy *	18.9		1b, 1e, 1f, 1k
16	FC	29	F	18	Epilepsy *	4.2	3.061	1b, 1e, 1f, 1k, 2a, 2b, 2c, S1a
17	FC	30	M	20	Healthy control	1.6	6.089	1b, 1e, 1f, 1k, 2a, 2b, 2c
18	FC	36	M	20	Healthy control	3.5	5.665	1b, 1e, 1f, 1k, 2a, 2b, 2c
19	FC	37	F	13	Healthy control	12.7		1b, 1e,1f, 1k
20	FC	38	M	17	Healthy control	9.8		1b, 1e, 1f, 1k
21	FC	39	M	23	Healthy control	14.3		1b, 1e, 1f, 1k
22	FC	39	M	14	Healthy control	24.1		1b, 1e, 1f, 1k, S1a
23	FC	42	M	18	Healthy control	26.6		1b, 1e, 1f, 1k
24	FC	43	F	22	Healthy control	0.7	4.601	1b, 1e, 1f, 1k, 2a, 2b, 2c, S1a
25	FC	44	F	21	Spina bifida *	0.0		1b, 1e, 1k, S1c
26	FC	49	F	16	Healthy control	9.8	5.622	1b, 1e, 1f, 1k, 2a, 2b, 2c, S3c
27	FC	49	F	14	Healthy control	3.3		1b, 1e, 1k
28	FC	54	M	16	Alzheimer disease	0.0		1b, 1e, 1f, 1k
29	FC	54	F	23	Huntington disease	7.6		1b, 1e, 1k
30	FC	55	F	16	Healthy control	6.7	5.829	1b, 1e, 1f, 1k, 2a, 2b, 2c
31	FC	56	M	23	Healthy control	12.6		1b, 1e, 1f, 1g, 1h, 1k, S1b
32	FC	57	M	n.d.	Healthy control	35.6		1e
33	FC	62	M	15	Brain hemorrhage *	8.4	6.525	1b, 1e, 1f, 1k, 2a, 2b, 2c, S1a
34	FC	65	M	5	Lewy body dementia	0.0	6.473	1e, 1j, 1k, 1l, 1m, 2a, 2b, 2c
35	FC	65	M	14	Sporadic Parkinson's	0.0	9.112	1e, 1k, 2a, 2b, 2c
36	FC	65	F	42	Healthy control	3.0	5.768	1a, 1b, 1e, 1k, 2a, 2b, 2c, S2e
37	FC	66	M	n.d.	Healthy control	3.3	6.674	1b, 1e, 1f, 2a, 2b, 2c, S1a
38	FC	68	M	17	Healthy control	0.0	2.514	1b, 1e, 1f, 1g, 1h, 1k, 2a, 2b, 2c, S1b
39	FC	70	F	n.d.	Healthy control	2.9	6.897	1b, 1e, 1f, 2a, 2b, 2c, S1a
40	FC	70	M	n.d.	Healthy control	4.6	5.459	1b, 1e, 1f, 1g, 1h, 2a, 2b, 2c, S1b
41	FC	72	M	n.d.	Lewy body dementia	28.4	6.274	1e, 2a, 2b, 2c
42	FC	75	M	48	Healthy control	32.5	2.878	1b, 1e, 1k, 2a, 2b, 2c
43	FC	75	F	13	Ischaemic stroke *	4.4		1b, 1e, 1f, 1k
44	FC	75	M	17	Lewy body dementia	0.0		1e, 1j, 1k
45	FC	76	M	74	Pick's disease	0.0		1e, 1k
46	FC	85	F	15	Alzheimer disease	0.0		1b, 1e, 1f, 1j, 1k
ARPD1	FC	66	M	17	*PRKN*-linked Parkinson’s	n/a		
ARPD2	FC	57	M	31	*PRKN*-linked Parkinson’s	n/a		
ARPD3	FC	70	F	42	*PRKN*-linked Parkinson’s	n/a		
ARPD4	FC	70	M	13	*PRKN*-linked Parkinson’s	n/a		
47	MB	26	M	2	Multiple sclerosis *			1b, S2b
65	MB	34	M	n.d.	Encephalitis*			1b
25	MB	44	F	21	Healthy control			1b
48	MB	44	F	5	Multiple sclerosis *			1c, S2b
49	MB	45	M	13	Ischaemic stroke *			1b
50	MB	47	F	20	Brain hemorrhage *			1b
51	MB	56	M	44	Multiple system atrophy			1b
52	MB	60	M	16	Progressive supranuclear palsy			1b
53	MB	61	M	20	Healthy control			1b
54	MB	61	M	3.5	Multiple sclerosis *			1b
36	MB	65	F	42	Healthy control			1b, S2a
55	MB	65	M	6	Multiple sclerosis *			1b
64	MB	71	M	n.d.	Lewy body dementia			1b
41	MB	72	M	n.d.	Lewy body dementia			1b
56	MB	74	F	n.d.	Amyotrophic lateral sclerosis			1b
42	MB	75	M	48	Healthy control			1b
57	MB	75	M	70	Sporadic Parkinson's			1b
45	MB	76	M	74	Pick's disease			1b
58	MB	79	M	n.d.	Progressive supranuclear palsy			1b
59	MB	82	M	48	Lewy body dementia			1b
60	SC/Muscle	68	F	2.5	Ischaemic stroke *			1c, 1d
61	SC/Muscle	64	F	2	Subarachnoid hemorrhage*			1d
62	SC/Muscle	74	M	2	Cerebellar hemorrhage *			1d
63	SC/Muscle	50	M	4	Subarachnoid hemorrhage *			1d

Supplementary Table 1: List of human tissue specimens examined in this study.
Characteristics listed include brain regions of frontal cortex (F ctx), midbrain, thoracic spinal cord (harvested with skeletal muscle); age (in years); sex (F, female; M, male); PMI, *post mortem* interval recorded in hours (hrs); n.d., not determined with accuracy (*i.e.*, inconsistent PMI information); brain diagnosis, where * indicates that the tissue examined was not affected by a detectable disease process; % parkin solubility (TS+TX/total signal); and the corresponding figure(s); S, supplementary data.

Supplementary Table 2: Cysteine residues in recombinant, human parkin are redox active.
Aliquots of human recombinant (r-) parkin that were oxidized by variable concentrations of H_2O_2 *vs.* control preparations were differentially labelled with iodoacetamide (IAA) and/or N-ethylmaleimide (NEM; as in Figure 3e) to identify reduced cysteines (IAA) or reversibly-oxidized residues (NEM). Proteins were subjected to LC-MS/MS and analyzed using Mascot Scaffold PTM to identify IAA (•) or NEM (+) adducts indicating when these were detectable on individual residues. Cysteines that were not detected as modified in individual runs are also listed (n/d). Note that cysteines within all four RING domains of human parkin as well as in the linker and UbL domains could be variably modified.

	**Treatment**	Control	Control	H_2O_2	H_2O_2	H_2O_2	H_2O_2	H_2O_2
	**Run**	IAA+NEM	IAA	20µM	1mM	4.5mM	4.5mM	4.5mM
**Region**	**Cysteine Residue**							
UBL	59		•	n/d	•	• +	•	•
Linker	95	•	•	•	•	• +	• +	• +
RING0	150	•	•	•	•	• +	• +	•
	154	•	•	n/d	•	• +	• +	•
	166	n/d	n/d	•	• +	n/d	•	•
	169	n/d	•	•	• +	n/d	•	•
	182	•	•	•	n/d	• +	• +	•
	196	•	•	•	•	• +	• +	• +
	201	•	•	•	•	• +	• +	• +
	212	•	•	•	n/d	• +	• +	• +
RING1	238	•	•	• +	•	• +	•	• +
	241	•	•	• +	•	• +	•	•
	253	•	•	• +	•	• +	•	•
	260	•	•	n/d	n/d	• +	•	•
	263	•	•	n/d	n/d	• +	•	•
	268	•	•	n/d	n/d	• +	•	•
	289	•	•	n/d	n/d	• +	• +	• +
	293	•	•	n/d	•	•	• +	• +
	323	•	•	n/d	n/d	• +	• +	• +
IBR	332	•	•	n/d	n/d	• +	•	•
	337	•	•	n/d	•	• +	•	• +
	352	•	•	•	•	•	• +	• +
	360	•	•	•	•	• +	•	•
	365	•	•	•	n/d	• +	•	•
	368	•	•	n/d	n/d	• +	• +	•
	377	•	•	•	n/d	• +	• +	• +
RING2	418	n/d	n/d	n/d	•	n/d	n/d	n/d
	421	n/d	n/d	n/d	• +	n/d	• +	• +
	431	n/d	•	n/d	•	n/d	•	•
	436	n/d	n/d	n/d	• +	n/d	•	•
	441	n/d	n/d	n/d	• +	n/d	•	•
	446	•	•	n/d	n/d	• +	• +	• +
	449	•	•	n/d	n/d	• +	• +	•
	451	•	•	n/d	n/d	• +	•	•
	457	•	•	•	•	• +	•	• +
	% Parkin protein coverage	83	89	57	60	86	98	97
	# peptides identified	40	35	22	33	38	51	47
	# Cysteines identified	27	31	16	21	28	34	34
	# IAA-cysteines	27	30	16	21	28	34	34
	IAA-cys/identified-cys	27/27	30/31	16/16	21/21	28/28	34/34	34/34
	%	100	97	100	100	100	100	100
	# NEM-cysteines	0	n/a	3	5	26	16	14
	NEM-cys/identified-cys	0/27	n/a	3/16	5/21	26/28	16/34	14/34
	%	0	n/a	19	24	93	47	41

• IAA + NEM
