# Supplementary material for: Age-associated insolubility of parkin in human midbrain is linked to redox balance and sequestration of reactive dopamine metabolites
Source: Acta Neuropathol. 2021 Mar 10;141(5):725–54. doi: 10.1007/s00401-021-02285-4 (PMC8043881; doi:10.1007/s00401-021-02285-4)
Supplement: Supplementary file 2 — Supplementary file2 (PPTX 7815 KB) [file 401_2021_2285_MOESM2_ESM.pptx]

## Slide 1
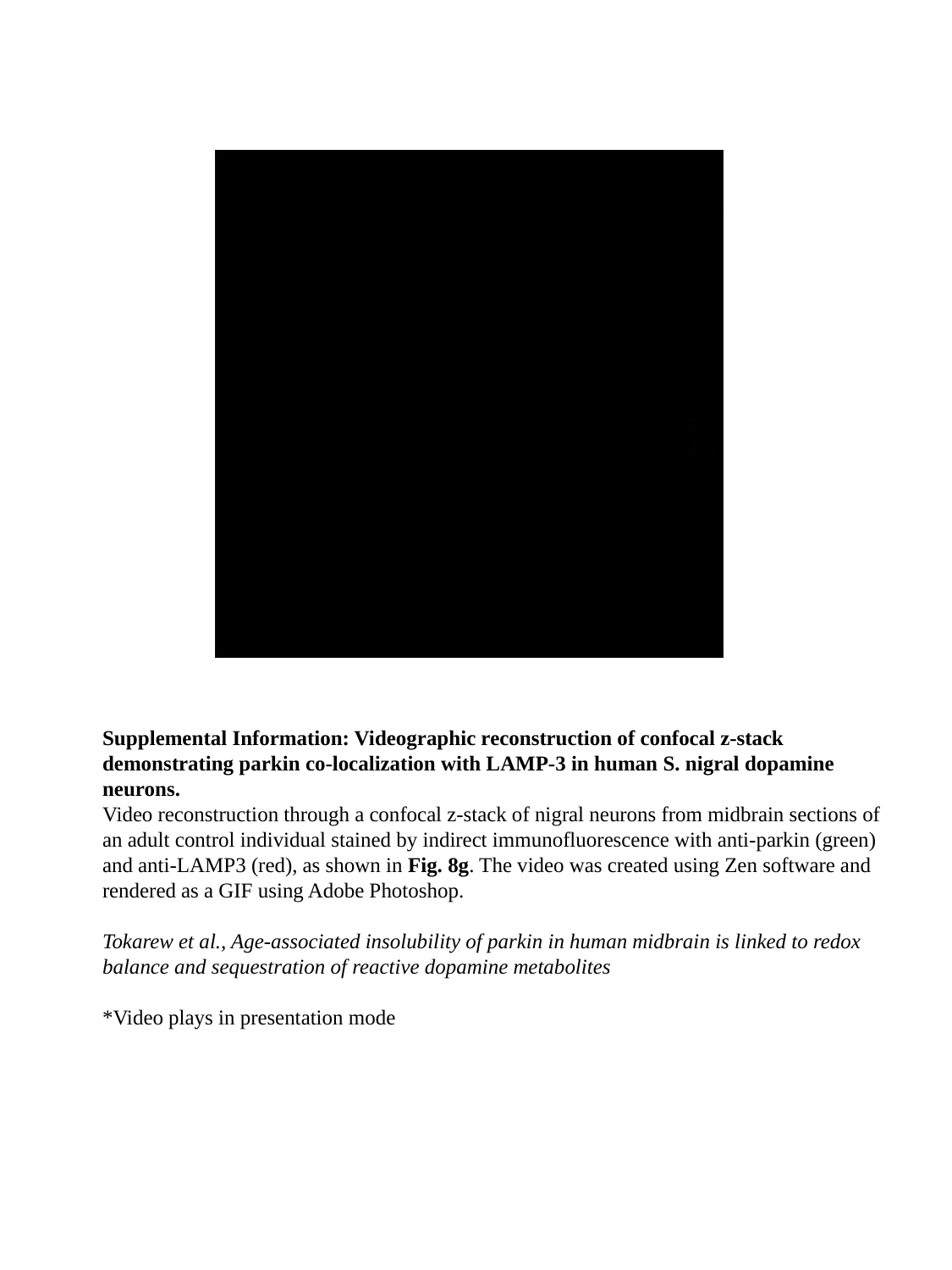

Supplemental Information: Videographic reconstruction of confocal z-stack demonstrating parkin co-localization with LAMP-3 in human S. nigral dopamine neurons.
Video reconstruction through a confocal z-stack of nigral neurons from midbrain sections of an adult control individual stained by indirect immunofluorescence with anti-parkin (green) and anti-LAMP3 (red), as shown in Fig. 8g. The video was created using Zen software and rendered as a GIF using Adobe Photoshop.
Tokarew et al., Age-associated insolubility of parkin in human midbrain is linked to redox balance and sequestration of reactive dopamine metabolites
*Video plays in presentation mode
